# Reverse Transcription Errors and RNA–DNA Differences at Short Tandem Repeats

**DOI:** 10.1093/molbev/msw139

**Published:** 2016-07-12

**Authors:** Arkarachai Fungtammasan, Marta Tomaszkiewicz, Rebeca Campos-Sánchez, Kristin A. Eckert, Michael DeGiorgio, Kateryna D. Makova

**Affiliations:** ^1^Integrative Biosciences, Bioinformatics and Genomics Option, Pennsylvania State University; ^2^Department of Biology, Pennsylvania State University; ^3^Center for Medical Genomics, Pennsylvania State University; ^4^Huck Institute of Genome Sciences, Pennsylvania State University; ^5^Department of Pathology, The Jake Gittlen Laboratories for Cancer Research, The Pennsylvania State University College of Medicine; ^6^Institute for CyberScience, Pennsylvania State University

**Keywords:** microsatellites, tandem repeats, RNA sequencing, RNA–DNA differences, transcription errors, reverse transcription errors, sequencing errors, error correction model.

## Abstract

Transcript variation has important implications for organismal function in health and disease. Most transcriptome studies focus on assessing variation in gene expression levels and isoform representation. Variation at the level of transcript sequence is caused by RNA editing and transcription errors, and leads to nongenetically encoded transcript variants, or RNA–DNA differences (RDDs). Such variation has been understudied, in part because its detection is obscured by reverse transcription (RT) and sequencing errors. It has only been evaluated for intertranscript base substitution differences. Here, we investigated transcript sequence variation for short tandem repeats (STRs). We developed the first maximum-likelihood estimator (MLE) to infer RT error and RDD rates, taking next generation sequencing error rates into account. Using the MLE, we empirically evaluated RT error and RDD rates for STRs in a large-scale DNA and RNA replicated sequencing experiment conducted in a primate species. The RT error rates increased exponentially with STR length and were biased toward expansions. The RDD rates were approximately 1 order of magnitude lower than the RT error rates. The RT error rates estimated with the MLE from a primate data set were concordant with those estimated with an independent method, barcoded RNA sequencing, from a *Caenorhabditis elegans* data set. Our results have important implications for medical genomics, as STR allelic variation is associated with >40 diseases. STR nonallelic transcript variation can also contribute to disease phenotype. The MLE and empirical rates presented here can be used to evaluate the probability of disease-associated transcripts arising due to RDD.

## Introduction

Transcription transfers genetic information from DNA to RNA, and multiple types of transcripts (e.g., transfer RNA, ribosomal RNA, messenger RNA, etc.) have critical functions in the cell. Therefore, the modifications or errors that occur in transcripts can lead to phenotypic variation among tissues and individuals. RNA–DNA differences (RDDs) are created by specific enzymatic machinery leading to RNA editing (Bass 2002; Schaub and Keller 2002), or arise as RNA polymerase errors during transcription (Blank et al. 1986; Ninio 1991; Strathern et al. 2012, 2013; Knippa and Peterson 2013; Zhou et al. 2013). RDDs increase the variability of transcripts and proteins. Note that RDDs can contribute to inherited variation in the sense that the enzymatic machinery responsible for RNA editing is genetically encoded (Gu et al. 2016). Several loci undergo RNA editing consistently in a large number of species (Corneille et al. 2000; Ibrahim et al. 2008; Danecek et al. 2012). In comparison with mutations, RDDs have lower evolutionary cost because an organism with RDDs can achieve higher phenotypic plasticity while retaining wild-type alleles (Gommans et al. 2009). As RDDs can enhance the adaptability of an organism to the environment, some level of RDDs is expected to be beneficial (Garrett and Rosenthal 2012a, 2012b; Rieder et al. 2015). However, a recent large-scale comparative genomics study found that, although some sites undergoing RNA editing might be under selective constraint (Xu and Zhang 2015), the majority of them do not have the characteristics of beneficial modifications (Xu and Zhang 2014).

With the availability of next-generation sequencing (NGS) data from whole genomes and transcriptomes of many different species, RDDs have been extensively studied, particularly with respect to base-substitution RNA editing (Li et al. 2009, 2011; Bahn et al. 2012; Bass et al. 2012; Pachter 2012; Park et al. 2012; Peng et al. 2012; Ramaswami et al. 2012, 2013). Moreover, RDDs arising from RNA editing were demonstrated in biochemical experiments. For example, adenosine deaminases can transform adenosine into inosine (which is read as guanine by a sequencing instrument) (Bass 2002; Schaub and Keller 2002), and apolipoprotein B mRNA editing enzymes can change cytosine into uracil (Wedekind et al. 2003). Other types of base-substitution RDDs have also been reported (Li et al. 2011). However, the extent to which technical and methodological errors contribute to RDDs is unclear (Bass et al. 2012; Pachter 2012). Some studies identified an excess of RDD sites toward the termini of NGS reads (Li et al. 2011; Kleinman and Majewski 2012; Pickrell et al. 2012), a location known to have high sequencing error rates (Kleinman and Majewski 2012; Pickrell et al. 2012), or in duplicated regions of the genome (Li et al. 2011; Ramaswami et al. 2012), in which RDDs can arise from a misalignment of paralogs (Schrider et al. 2011; Kleinman and Majewski 2012; Lin et al. 2012). Besides RNA editing, transcription errors leading to base-substitution RDDs were also studied and were found to exhibit similar rates across different types of transcripts and growth states of bacteria (Traverse and Ochman 2016).

RDDs in the form of insertions and deletions, particularly at short tandem repeats (STRs), have been less studied than base-substitution RDDs. Indeed, RNA editing that expands or contracts STRs is yet to be demonstrated. Transcription errors at STRs have been shown both in vitro and in vivo (Strathern et al. 2012, 2013; Zhou et al. 2013). Although the frequency with which such errors occur has not been evaluated quantitatively, possessing some fraction of malfunctioning RNA is expected to have a smaller effect on the fitness of an organism in comparison with malfunctioning DNA, which could affect the cells and body throughout a lifetime and is transmitted to daughter cells in the case of a germ-line mutation.

STRs, which after a certain number of repeats are also called microsatellites (Kelkar et al. 2010; Ananda et al. 2013), exhibit high mutation rates due to polymerase slippage (Drake et al. 1998; Ellegren 2000; Vigouroux et al. 2002; Ellegren 2004; Baptiste et al. 2013, 2015). They are particularly important for understanding disease susceptibility, as mutations at STRs are implicated in over 40 neurological disorders (Boby et al. 2005; Pearson et al. 2005; Castel et al. 2010), and, more than 30% of human genes contain one or more STRs in their exonic regions (Legendre et al. 2007). All classes of long STRs have been found to be overrepresented in disease-associated genes (Madsen et al. 2008), and some relatively short STRs have also been implicated in disease. For example, a (CGC)_*n*_ repeat number change from *n* = 11 to *n* = 12 in the *PABPN1* gene can cause Oculopharyngeal Muscular Dystrophy (Brais 1998). As RDDs at a locus with a wild-type allele can result in a transcript that mimics a transcript from a disease-causing allele, STR RDDs may have pathological consequences. Thus, estimating RDD rates at STRs is critical for understanding the fidelity of transcription, and for estimating the probability of disease occurrence as a consequence of transcript alteration. If the estimated STR RDD rates are high, then this observation can significantly change the paradigm of medical genomics in the diagnostics of diseases caused by STR mutations.

Detecting RDDs at STRs is challenging for a number of reasons. First, conventional short-read mapping approaches favor alignments to the reference allele (Gymrek et al. 2012; Fungtammasan et al. 2015) and, as a result, the transcription error rates can be underestimated. Second, short-read sequencing at STRs is error-prone (Ross et al. 2013; Fungtammasan et al. 2015) and sequencing errors can be misinterpreted as transcription errors. These two limitations can be alleviated with the use of the STR-FM pipeline, which incorporates flank-based mapping and utilizes previously estimated STR sequencing error rates (Fungtammasan et al. 2015). Third, the profile and rates of reverse transcription (RT) errors at STRs are unknown. If these rates are high, then they can greatly affect the estimation of RDDs. Thus, it is crucial to consider RT errors in STR RDD studies. Fourth, STRs are highly mutable and exhibit substantial somatic and inter-individual genetic variation (O’Huallachain et al. 2012). This somatic variation can lead to STR length variation among tissues. Therefore, to accurately detect STR RDDs, it is necessary to study DNA and RNA from the same tissue of the same individual.

Recently, the barcoded RNA sequencing technique (Gout et al. 2013) was proposed as an approach for studying RDD and RT errors. In this technique, each RNA molecule is tagged with a unique barcode, which makes it possible to trace all subsequent cDNA molecules and sequencing reads. In combination with several rounds of cDNA library construction from the same set of barcoded RNA, the consensus cDNA and RNA sequences can be generated, and the RDD and RT error rates can be estimated based on the proportion of incongruent reads.

Although barcoded RNA sequencing is a powerful technique for estimating RDD and RT error rates, an alternative approach would still be useful. On the one hand, there is a need to estimate these rates from the existing data sets not processed with RNA barcoding. Such data sets are highly abundant and will allow the reliable estimation of RT error and RDD rates at STRs that require ample data for their analysis because of the flank-based mapping (Gymrek et al. 2012; Fungtammasan et al. 2015). The existing barcoded RNA data sets are currently of limited scale (Gout et al. 2013), and generating larger data sets is expensive. On the other hand, batch effects are inevitable, and different library preparation procedures can greatly affect RT error rates (Quail et al. 2012). A novel method to estimate RDD and RT error rates that is compatible with the standard method of RNA sequencing would be indispensable for correcting for batch effects.

To estimate RT error and RDD rates at STRs, we developed a maximum-likelihood estimator (MLE) that utilizes sequencing data from replicate cDNA libraries. Our method can be employed with conventional RNA sequencing procedures, and as such represents an attractive alternative to barcoded RNA sequencing. Using our method, we addressed three questions. First, what are the levels of RDDs and of RT errors at STRs, and do they exhibit contraction or expansion biases? To address this question, we generated DNA and RNA sequencing data from the same tissue of the same individual to eliminate the effects of somatic genetic variation, and simultaneously estimated RT error and RDD rates at STRs. Second, what are the precision and accuracy of our estimates? To assess these properties, we validated the estimated rates with a replicated trial and compared them with those obtained from the published barcoded RNA sequencing data (Gout et al. 2013). Finally, what are the RT error and RDD rates compared with the germ-line mutation rates and sequencing error rates at STRs? To evaluate these levels, we contrasted the RT error and RDD rates estimated here with published germ-line mutation rates and sequencing error rates (Sun et al. 2012; Fungtammasan et al. 2015).

## Results

### Experimental Design

**Fig. 1:**
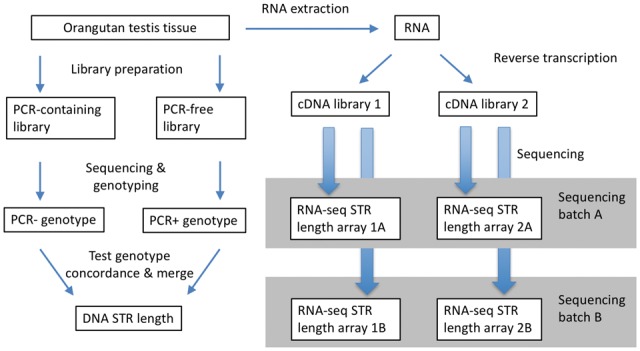
A schematic representation of the experimental design.

To study the RT error and RDD rates at STRs, we designed the following experiment (fig. 1). We isolated genomic DNA and total RNA from the same sample (orangutan testis of a single individual). The genomic DNA was sequenced using two different library preparation protocols—PCR-containing and PCR-free (see Materials and Methods section for details)—allowing us to test for genotype congruence between the two libraries (see “Genotyping STRs Using the DNA Sequencing Data” in Results). Total RNA was divided into two aliquots that were used to construct two separate RNA-seq libraries. Each of these two libraries was sequenced in two separate batches. Such an experiment, ideally, should allow one to differentiate between RDDs (such differences from the DNA sequence should be present in both RNA-seq libraries) and RT errors (such variants should be present in only one of the two RNA-seq libraries but in both sequencing batches). However, empirical data frequently have missing information at some loci due to limited sampling, which can distort results. For example, if a deviant STR variant is not sampled in one cDNA library, then an RT error can be incorrectly inferred instead of an RDD. For instance, if one-tenth of RNA molecules at a locus was modified from (A)_6_ to (A)_7_ due to RDD, then we should expect to observe (A)_7_ in both replicated cDNA libraries sequenced. However, if (A)_7_ was not sampled in one library, then we will observe (A)_7_ only in the other library, thereby misclassifying this situation as an RT error. Therefore, we developed a full likelihood method that permits sampling errors in the likelihood calculation to avoid error misclassifications.

The rationale behind the method is in the correlation of variants observed between cDNA libraries. RDDs lead to correlated shifts in the distribution of variants between cDNA libraries, whereas RT errors lead to independent shifts between these distributions. For example, suppose at a locus the repeat number in the DNA is *D*, and we observe variants with repeat lengths *D* − 1 at high frequency in one cDNA library and *D *+ 1 at high frequency in another cDNA library. Such a scenario is likely to have occurred due to substantial RT errors at this locus, as a large portion of the distribution is different between the libraries. However, now suppose that we instead observe variants with repeat length *D *+ 1 at high frequency in both cDNA libraries. Such a scenario could have occurred through either RDDs or RT errors, though this probability is lower for RT errors than for RDDs, with the uncertainty accounted for within our likelihood method. Finally, suppose that we instead observe variants with repeat length *D *+ 2 at high frequency in both cDNA libraries. Such a scenario is likely to have occurred due to both substantial RDDs and RT errors, because at each step the stepwise mutation model only permits a change in the STR repeat length by one unit. By taking the likelihood across independent loci, we are accumulating evidence for the prevalence of each scenario, and are also directly accounting for the uncertainty by modeling the unobserved states (RNA and actual cDNA).

### Genotyping STRs Using the DNA Sequencing Data

Sequencing of the PCR-containing and PCR-free genomic DNA libraries resulted in the estimated genome-wide mean sequencing depth of 6.7× (267 million reads) and 1.8× (73 million reads), respectively. We employed our previously published software, STR-FM (Fungtammasan et al. 2015), to locate STRs in DNA sequencing reads. Namely, STRs with at least five mono-, three di-, three tri-, and three tetranucleotide repeats were detected in reads from each sequenced library (see Materials and Methods). After mapping such reads to the orangutan reference genome, we utilized published sequencing error rates (Fungtammasan et al. 2015) to genotype STRs at each locus. To estimate genotyping accuracy, we used loci for which we could derive genotypes from both libraries. For them, the genotypes from the PCR-free library were compared with those from the PCR-containing library. This comparison resulted in a 99.86% genotype concordance (supplementary table S1, Supplementary Material online), a higher concordance than that achieved in previous studies (Gymrek et al. 2012; Fungtammasan et al. 2015). After removing discordant genotypes, we merged the data from the two libraries and limited our analysis to homozygous loci (supplementary table S1, Supplementary Material online) to reduce complexity of MLE estimation (also see “Samples, DNA Sequencing, and Genotyping” in Materials and Methods). They constitute 99.5% of our data. After additional filtering (see “Samples, DNA Sequencing, and Genotyping” in Materials and Methods), we retained 5,582,009 mono-, 2,768,451 di-, 309,546 tri-, and 78,454 tetranucleotide STR-containing loci.

### STR Profiling of RNA

For the RNA-seq data, we generated a total of 56.6, 55.3, 39.7, and 38.9 million paired-end reads for library 1 batch A, library 2 batch A, library 1 batch B, and library 2 batch B, respectively. These sequencing depths are higher than those recommended by the best practice guidelines for gene expression studies of species with a reference genome (ENCODE 2011; Conesa et al. 2016). Though our MLE does not require that sequencing depth is balanced among cDNA libraries, we chose to balance the sequencing depths to avoid any unforeseen biases. To balance the depths, we downsampled library 1 batch A to 55.3 million reads and library 1 batch B to 38.9 million reads to have the equivalent number of reads between the two libraries sequenced in the same batch.

To profile STRs in the RNA-seq data, we followed the same procedure as that used for DNA data. Briefly, STR-containing RNA-seq reads were mapped to the reference genome, and reads with uniquely mapping flanking sequences (20 bp upstream and 20 bp downstream from an STR) were retained. This procedure resulted in length profiles (a collection of lengths from the reads mapping to this locus) for each STR locus. Each RNA-seq library and each sequencing batch was analyzed separately (fig. 1).

We focused our analysis on STRs with the (A/T)_*n*_ motif ((A/U)_*n*_ for RNA). We call this motif (A)_*n*_ for brevity. Most analyses were performed in the range of (A)_5_–(A)_10_ because of the high abundance of STRs with this repeat number (supplementary table S2, Supplementary Material online) (Subramanian et al. 2003), and due to their high propensity to polymerase slippage (Ellegren 2000, 2004; Ananda et al. 2011; Fungtammasan et al. 2015). Other motifs are discussed in the “Estimation of STR RDD and RT Error Rates Using MLE” subsection of Results. Overall, in the RNA-seq data, the number of loci with the (A)_*n*_ motif decreased as the STR length increased (supplementary fig. S6, Supplementary Material online), which is expected based on the distribution of STRs in the genome (Denver et al. 2004; Fungtammasan et al. 2015). For each STR length, the (A)_*n*_-containing loci with low expression level (proxied by the number of RNA-seq reads per locus) were considerably more prevalent than the loci with high expression level (supplementary fig. S2, Supplementary Material online). Thus, most (A)_*n*_-containing loci in our data set were short and had low expression levels.

### An MLE to Estimate RT Error and RDD Parameters

To estimate the RT error and RDD rates and their expansion probabilities, we developed an MLE that jointly infers this set of parameters by maximizing the likelihood of observing a given set of sequenced STR length profiles. Although the model includes expansion probabilities for RT errors and for RDDs, the corresponding contraction probabilities can be computed as one minus the expansion probability in each case. The model requires one DNA data set and a minimum of two replicated RNA-seq data sets from the same sample. For the observed read data that originated from the same STR motif and length (e.g., (A)_7_), our method calculates the likelihood of the data being generated from all possible combinations of RNA forms and all possible combinations of cDNA forms given the set of four parameters (RT error rate, RT expansion probability, RDD rate, and RDD expansion probability). By identifying the parameter set that results in the highest likelihood value, our model makes use of the replicated cDNA library structure (fig. 1) to enhance our ability to distinguish between RT errors and RDDs.

### Performance of MLE

To evaluate the ability of the MLE to infer the four parameters of interest, we conducted simulations using several sets of model parameters, numbers of loci, and bin sizes. The bin size is the number of sampled molecules at the RNA or cDNA stages, which determines the set of possible distinct STR length distributions for RNA and cDNA. This bin size affects the sampling process of each cDNA library from an RNA sample and each RNA sample from the DNA sample. Small bin sizes will yield a high sampling error, leading to distortions in the distribution of RNA or cDNA STR forms relative to the distribution expected under the stepwise mutation model. The results of the simulations indicate that the MLE can estimate all four parameters with a high level of precision and accuracy (supplementary figs. S3, S4, and S7–S10, Supplementary Material online), provided certain conditions are met. First, the chosen bin size *M* must be close to the number of reads per locus of the RNA-seq data, proxying gene expression level (supplementary figs. S3, S4, and S7–S10, Supplementary Material online). Although the optimal combination between bin size and the number of RNA-seq reads per locus varies among parameter sets (supplementary figs. S3, S4, and S7–S10, Supplementary Material online), the MLE performs reasonably well when the number of RNA-seq reads per locus is between *M* and 2*M.* For example, the estimated RT error and RDD rates for a bin size of 2 are the most accurate when the simulated data were generated using three molecules of RNA and three molecules of cDNA (supplementary figs. S3 and S4, Supplementary Material online). Second, the number of loci must be at least the inverse of the error rates. The higher the number of loci, the more accurate the estimates. When both conditions are met, the true parameters are bound by 95% of the estimated parameters, and the median estimates deviate from the true parameters by less than 10% (supplementary figs. S3, S4, and S7–S10, Supplementary Material online).

### Lumping MLE

Because the optimal bin size for MLE increases with the number of RNA-seq reads per locus (expression level), it is computationally challenging to estimate RT error and RDD rates from loci expressed at high levels. Therefore, we developed an approximation to the MLE, which we call the *lumping MLE*, that substantially reduces the number of calculations in the likelihood (supplementary text S2, Supplementary Material online) as compared with that in our original, or “*full, MLE*” (see “MLE Formulation” in Materials and Methods). We validated this method using loci expressed at low levels and compared its results with those obtained using the full MLE. The parameter estimates and their corresponding 95% confidence intervals of the same data sets are strikingly similar between the full and lumping MLE methods (supplementary table S3, Supplementary Material online). For example, at a bin size of 5, both the full MLE and the lumping MLE can estimate RDD rates for the data with ten RNA-seq reads per locus with less than 5% error. Because of a similar performance but applicability to a larger range of expression levels than for the full MLE, we will use the lumping MLE to estimate RT error and RDD rates for STR loci with six or more RNA-seq reads per locus.

### Estimation of the STR RT Error and RDD Rates Using MLE

**Fig. 2:**
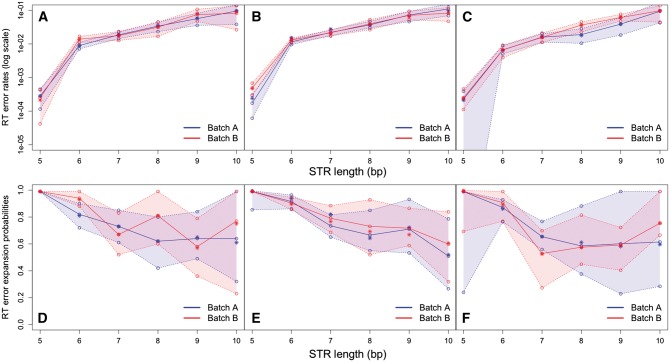
A comparison of RT error rates and RT expansion probabilities as a function of repeat number for motif (A)_*n*_ between sequencing batches A (blue) and B (red). (*A*) RT error rates for the bin size of 2; (*B*) RT error rates for the bin size of 5; (*C*) RT error rates for the bin size of 40; (*D*) RT expansion probabilities for the bin sizes of 2; (*E*) RT expansion probabilities for the bin size of 5; (*F*) RT expansion probabilities for the bin size of 40. Repeat numbers between 5 and 10 were chosen due to their high abundance. Median values across 100 empirical bootstrap replicates (bootstrapped across loci) are plotted with open circles, whereas point estimates are plotted with stars. Solid lines connect the median bootstrap estimates. The 95% confidence intervals were calculated from the 100 bootstraps replicates. Each estimate was based on five sets of random initial parameters to minimize the possibility of reaching local maxima, and the set of parameters that had the maximal likelihood was taken as the estimate for a given bootstrap replicate. The estimations for the bin size of 2 were performed using full MLE, whereas the estimations for the bin size of 5 and 40 were performed using lumping MLE. The number of loci analyzed for each bin size is listed in supplementary tables S4 and S5, Supplementary Material online.

Using the full MLE and the bin size of 2, we first estimated the RT error and RDD rates, as well as RT error and RDD expansion probabilities, at (A)_*n*_-containing loci expressed at low levels (i.e., with three to five RNA-seq reads per locus). The exceptionally low RT error rate for repeat (A)_5_ was most likely due to our detection threshold for mononucleotide STRs—we only collected such STRs starting from five repeats and thus could not observe RT errors (and RDDs) that changed (A)_5_ to (A)_4_. As the repeat number increased from 6 to 9 bp, the RT error rates increased exponentially from 2.1 × 10^−^^4^ to 7.7 × 10^−^^2^, (fig. 2A; supplementary table S4, Supplementary Material online). Our estimates of RT error rates had narrow 95% confidence intervals and were highly similar between the two sequencing batches (blue and red lines in fig. 2A). The RT errors from (A)_6_ to (A)_9_ exhibited an expansion bias (fig. 2D). The expansion bias decreased as the repeat number increased (fig. 2D and supplementary table S4, Supplementary Material online); the 95% confidence intervals also widened because the number of loci evaluated decreased (second column in supplementary table S4, Supplementary Material online). Similar to the pattern observed for RT errors, RDD rates increased with STR length (table 1). However, the RDD rates were substantially lower than the RT error rates (table 1 and supplementary table S4, Supplementary Material online, fig. 2A). For example, the average RT error rate between the two batches at (A)_8_ was 3.7 × 10^−^^2^, whereas the average RDD rate at the same repeat number was 4.2 × 10^−^^3^. Because RDD rates were rather low, we could not estimate them for several repeat numbers (as we lacked sufficient data to detect such low rates), and the 95% confidence intervals for those that we could estimate were wide (supplementary table S4, Supplementary Material online). The same is true for our estimates of RDD expansion probability (supplementary table S4, Supplementary Material online).

**Table 1:** RDD Rates for the (A)_*n*_ Motif.

	**Bin = 2; 3–5 RNA-seq Reads**		**Bin = 5; 6–16 RNA-seq Reads**		**Bin = 40; 49–102 RNA-seq Reads**	
	Batch A	Batch B	Batch A	Batch B	Batch A	Batch B
**(A)_5_**	<1.0e-9 [<1.0e-9, <1.0e-9]	<1.0e-9 [<1.0e-9, 2.76e-4]	<1.0e-9 [<1.0e-9, 3.29e-9]	<1.0e-9 [<1.0e-9,6.86e-5]	1.87e-4 [<1.0e-9, 5.70e-4]	<1.0e-9 [<1.0e-9, 4.29e-9]
**(A)_6_**	1.87e-3 [2.45e-4, 3.38e-3]	5.13e-4 [<1.0e-9, 2.01e-3]	6.45e-4 [<1.0e-9, <2.26e-3]	6.80e-4 [<1.0e-9, 2.17e-3]	1.89e-3 [<1.0e-9, 3.42e-3]	1.81e-3 [<1.0e-9, 4.97e-3]
**(A)_7_**	<1.0e-9 [<1.0e-9, 2.28e-3]	2.59e-3 [<1.0e-9, 7.59e-3]	3.26e-3 [<1.0e-9, 2.34e-3]	3.9e-3 [<1.0e-9, 2.83e-3]	7.36e-4 [<1.0e-9, 5.36e-3]	5.90e-3 [<1.0e-9, 1.33e-2]
**(A)_8_**	3.57e-3 [<1.0e-9, 1.48e-2]	2.68e-3 [<1.0e-9, 1.72e-2]	7.53e-3 [3.80e-3, <1.0e-9]	7.46e-3 [<1.0e-9, 1.14e-2]	3.76e-3 [<1.0e-9, 1.36e-2]	<1.0e-9 [<1.0e-9, 8.12e-3]
**(A)_9_**	8.94e-3 [<1.0e-9, 2.28e-2]	<1.0e-9 [<1.0e-9, <1.0e-9]	2.40e-2 [<1.0e-9, 1.90e-2]	1.57e-2 [<1.0e-9, 1.67e-2]	2.33e-2 [<1.0e-9, 7.34e-2]	<1.0e-9 [<1.0e-9, 1.49e-2]
**(A)_10_**	<1.0e-9 [<1.0e-9, 6.55e-2]	1.15e-2 [<1.0e-9, 8.22e-2]	7.52e-2 [<1.0e-9, 6.85e-2]	4.95e-3 [<1.0e-9, 3.54e-2]	7.54e-3 [<1.0e-9, 5.93e-2]	<1.0e-9 [<1.0e-9, 1.18e-2]

Note.—In each cell, the number outside the brackets is the point estimation, whereas the numbers inside the brackets are the 95% confidence intervals.

To confirm that our estimates were not affected by data selection or sequencing artifacts at loci expressed at low levels, we repeated the analysis with different bin sizes and ranges of expression level. We applied the lumping MLE to the data with the numbers of RNA-seq reads ranging from 6 to 16 (supplementary table S5, Supplementary Material online) using the bin size of 5 (fig. 2B and E), and from 49 to 102 using the bin size of 40 (fig. 2C and F). This range of RNA-seq read numbers does not overlap with the one used for the bin size of 2 (see “Estimation of RDD and RT Errors Using the MLE” in Materials and Methods) and thus provides an opportunity to estimate the parameters independently, but for the same sequencing batches. The resulting estimates of RT error rates and of RT error expansion probability were strikingly similar to those calculated based on the smaller number of RNA-seq reads and the bin size of 2 (fig. 2A–F). The RDD rates estimated using three different bin sizes all increase with repeat number; however, their more detailed comparison is challenging because of wide confidence intervals (table 1).

Our MLE can be applied to more than two replicated RNA sequencing data sets (see supplementary text S3, Supplementary Material online, for equation). For example, we simultaneously analyzed both cDNA libraries 1 and 2 for both batches A and B (four sequencing data sets) with lumping MLE with bin size of 5. The estimated RT error and RDD rates (supplementary fig. S11, Supplementary Material online) are similar to those obtained after analyzing batches A and B separately (fig. 2).

We also attempted to estimate RT error and RDD rates for other STR motifs. However, the numbers of loci were insufficient to estimate these rates accurately (supplementary table S2, Supplementary Material online). For example, the next most abundant group of STRs in our data after the (A)_*n*_-containing STRs were (AC)_*n*_- and (AG)_*n*_-containing STRs (supplementary table S2, Supplementary Material online). Among them, we identified only 5,142 loci with three to five RNA-seq reads per locus in batch A (supplementary table S6, Supplementary Material online). For such loci (combined for these two motifs), we only detected one deviant STR form at the consensus repeat number of 4 (one locus contained two reads of (AG)_3_), and inferred RDD rate of 0 and RT error rate of 5.84 × 10^−^^4^ (95% confidence interval from < 1.0 × 10^−^^9^ to 1.76 × 10^−^^3^) (supplementary table S7, Supplementary Material online). The RT error expansion probability was inferred to be 0, indicating a contraction bias; however, the 95% confidence interval was wide (supplementary table S7, Supplementary Material online). We conclude that we presently lack a sufficient amount of data to accurately evaluate the RT error and RDD rates at STRs others than (A)_*n*_.

### RDD and RT Error Rates Estimation Using Barcoded RNA Sequencing

To validate the MLE, we analyzed publicly available *C. elegans* barcoded RNA data (Gout et al. 2013) and evaluated RT error and RDD rates with an independent method, that is, barcoded RNA sequencing. According to this method, RNA molecules are tagged, allowing a direct inference of RDD rates by tracing cDNA molecules and sequencing reads that originated from the same RNA molecule (i.e., from the same “family”; supplementary fig. S5, Supplementary Material online). In the barcoded RNA data, using the modified STR-FM (Fungtammasan et al. 2015), we detected a total of 9,074,690 STR-containing cDNA reads (5,574,030 mono-, 2,455,300 di-, 21,018,578 tri-, and 26,782 tetranucleotide containing cDNA reads), based on which we inferred a total of 949,826 STR-containing cDNA molecules (supplementary table S8, Supplementary Material online). Because most of the cDNA families were present in just one of the three RNA-seq libraries (Gout et al. 2013), we could infer STR lengths in only 7,922 STR-containing RNA molecules (with 4,596 mono-, 1,376 di-, 1,948 tri-, and two tetranucleotides; supplementary table S9, Supplementary Material online). No errors were detected among the reads for each RNA molecule allowing us to estimate only maximal RDD rate as one divided by the number of loci for a specific motif (e.g., 1/3,549 for (A)_5_; supplementary table S9, Supplementary Material online).

For this data set, the barcoded RNA was also reverse transcribed (and sequenced) independently three times, allowing one to infer RT error rates. We found that all RT errors occurred at the (A)_*n*_-containing motif and that the RT error rates increased with increasing repeat number (supplementary table S10, Supplementary Material online). The 12 erroneous reads stemmed from RNA families with only two reads, and so we could not immediately determine whether these were expansion or contraction errors. However, based on the consensus repeat numbers of all reads mapped to these loci, we concluded that the RT errors had a preference toward expansions (eight expansions vs. four contractions; supplementary table S10, Supplementary Material online).

**Fig. 3:**
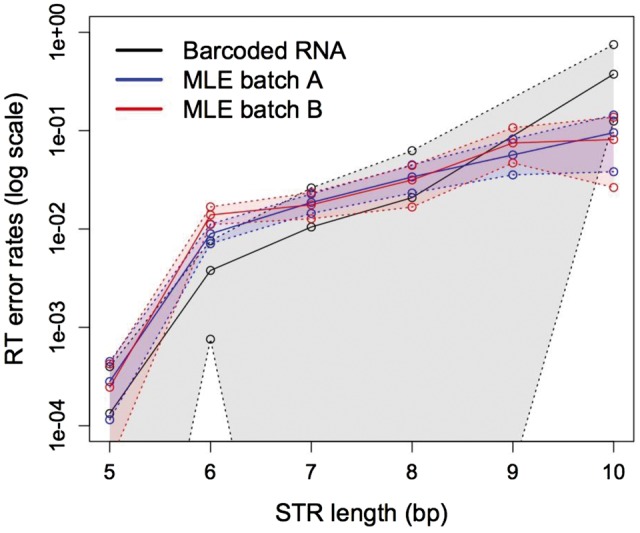
A comparison of RT error rates estimated using the full MLE (orangutan data) versus barcoded RNA sequencing (*Caenorhabditis elegans* data). The 95% confidence intervals for the rates estimated with the full MLE were generated from 100 empirical bootstrap replicates (bootstrapped across loci), whereas the 95% confidence intervals for the barcoded RNA sequencing were generated from 1,000 bootstrap replicates of inferred cDNA molecules with at least two cDNA molecules in that family. The lower bounds of the RT error rate confidence intervals for the barcoded RNA sequencing are zero and thus are outside the plotting area.

Notably, the point estimates of RT error rates were remarkably concordant between the *C. elegans* data set (where they were inferred with the RNA barcoded approach) and orangutan data set (where they were inferred with the MLE approach; fig. 3), even though the *C. elegans* data were more limited in scale and thus the estimates from it had wide confidence intervals. This concordance is particularly exceptional given that the rates were inferred by two different methods and from two independent data sets generated in two different laboratories (Gout et al. 2013). Indeed, the RT error rates estimated from the orangutan data using MLE and from the *C. elegans* data using barcoded RNA increased with increasing repeat numbers and their confidence intervals overlapped (fig. 3). The maximal RDD rate for *C. elegans* (supplementary table S9, Supplementary Material online) appears to be higher than but overall is comparable to the RDD estimates for orangutan (table 1 and supplementary table S4, Supplementary Material online). Because we can only estimate the maximal RDD rate from the *C. elegans* data (as one over the number of studied loci), we are not in a position to compare it with the RDD rate obtained from the orangutan data rigorously.

To additionally test the performance of our MLE, we applied the lumping MLE to the *C. elegans* data (Gout et al. 2013) after removing the barcodes, and estimated the RT error rates for this data set (supplementary fig. S12, Supplementary Material online). The resulting RT error rate estimates for the *C. elegans* data set were strikingly similar between the lumping MLE and barcoded RNA methods. This validates the use of the MLE method for reliable RT error rate estimation.

## Discussion

### Application of Estimated RT Error Rates

Knowing RT error rates can be instrumental in functional genomics analysis where RNA-sequencing data are one of the most important sources of information. RNA-sequencing data have been used to study differences in gene expression among tissues (GTEx Consortium et al. 2015), between samples of healthy and diseased individuals, and among organisms inhabiting various environments (Wang et al. 2009; Wilhelm and Landry 2009; Oshlack et al. 2010; Garber et al. 2011; Ozsolak and Milos 2011; McCarthy et al. 2012). Such data can also be utilized to study biological pathways (Feng et al. 2012; Khatri et al. 2012; Trapnell et al. 2013), metabolic flux (Gowen and Fong 2010; Lee et al. 2012), and individual health (Chen et al. 2012). Without estimating RT error rates, it is challenging to quantify expression level for such genes accurately. For example, if the RT error rate is high, then a large number of STRs in cDNA will vary in length, thereby reducing their mappability to the reference genome, which can lead to an underestimation of expression levels of genes containing STRs. Removing STR-containing regions can alleviate this problem, but will lead to an underestimation of true variation at the level of RNA.

Applications of RT error rates are not limited to functional genomics. STRs have been widely used as markers in population genomics due to their high polymorphism level (Wright and Bentzen 1994; Gupta and Varshney 2000; Sunnucks 2000; Miah et al. 2013; Abdul-Muneer 2014). According to a recent study (Gayral et al. 2013), RNA-sequencing can be applied to study population genomics of nonmodel organisms without a reference genome (De Wit et al. 2012; Gayral et al. 2013). (For model organisms with reference genomes, exome sequencing data are usually used as an alternative [DaRe et al. 2013; Guo et al. 2013; Samuels et al. 2013; Griffin et al. 2014]). It is crucial, however, to take into account such errors in order to distinguish genetic variation from technical errors.

### Relative Rates and Patterns of RDD, RT Errors, and Mutations at STRs

**Fig. 4:**
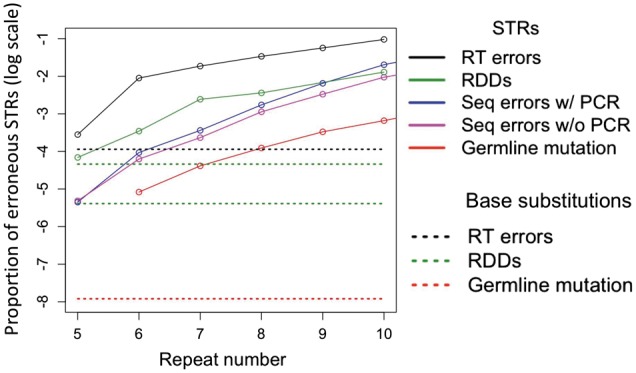
A comparison among STR RT error rates (this study), STR RDD rates (this study), STR germ-line mutation rates (Fungtammasan et al. 2015), STR sequencing error rates (Fungtammasan et al. 2015), base-substitution germ-line mutation rates (Kong et al. 2012), and base-substitution RDD rates (Gout et al. 2013 [lower line], Traverse & Ochman 2016 [upper line]).

The RDD rates obtained here provide the first opportunity to understand the propensity of STRs to increase in repeat number not only at the level of DNA but also at the level of RNA. For (A)_*n*_-containing STRs, we found that the RDD and RT error rates increase exponentially with repeat number—a pattern similar to that previously identified for germ-line mutations (Sun et al. 2012; Fungtammasan et al. 2015) and sequencing errors (Ross et al. 2013; Fungtammasan et al. 2015). This similarity can be explained by the increased propensity of polymerase slippage with an increase in the STR repeat number. Moreover, we inferred the RT error rates to be higher than the RDD rates, which were higher than the sequencing error rates (with the minimal Phred sequencing quality of 20), which, in turn, were higher than the germ-line mutation rates for STRs (fig. 4) (Kong et al. 2012; Gout et al. 2013; Fungtammasan et al. 2015). For mononucleotides with repeat numbers of 6 and 7, these differences were approximately 1 order of magnitude in size. For SNPs, the RT error rate (Gout et al. 2013) is also higher than the RDD rate (Gout et al. 2013; Traverse and Ochman 2016). Critically, the level of technical errors is higher than the level of biological errors. Therefore, accurate inferences of germ-line mutations must consider sequencing errors, and accurate estimations of RDD rates must consider RT errors.

Regarding technical errors, the most commonly used reverse transcriptase in molecular biology applications is the Moloney murine leukemia virus RT (MMLV-RT). The MMLV-RT enzyme has an in vitro error rate of 1/29,000 nucleotides synthesized using an RNA template, and 1/37,000 nucleotides using a DNA template, as determined using a genetic reporter assay (Ji and Loeb 1992). Although the majority of MMLV-RT errors are base substitutions, a mutational hotspot of one-base indels within an (A)_4_ sequence has been reported (Barrioluengo et al. 2011). Many protocols for generating cDNA, including the Illumina TruSeq RNA library preparation, use a modified version of MMLV-RT known as Superscript II reverse transcriptase. The Superscript II RT has improved thermostability but reduced fidelity, with an error rate of 1/15,000 nucleotides synthesized using a DNA template (Azeri and Hogrefe 2007). These reported MMLV-RT error rates are of similar magnitude to error rates measured for the Taq polymerase (∼1/10,000–1/50,000) for proofreading-proficient thermostable polymerases, measured using the same in vitro assay (Eckert and Kunkel 1990, 1991). Therefore, cDNA synthesis and sequencing error rates are not expected to vary substantially, unless a thermostable DNA polymerase with a highly efficient proofreading activity is used. Importantly, the extent to which the accuracy of MMLV-RT, Taq or proofreading-proficient thermostable polymerases will vary when copying longer STRs remains to be determined.

### The Reliability of the Estimates

The RT error rates we obtained were congruent between two independent methods—MLE and barcoded RNA sequencing—and between two independent data sets—the one obtained from an orangutan sample and the one obtained from the *C. elegans* sample—produced in two separate laboratories. This concordance suggests that our estimates are reliable.

We followed several procedures to control for technical errors that could distort RT error and RDD rates. We used the same tissue sample for both DNA and RNA sequencing to prevent somatic variation among different tissues. We genotyped the sample with two separate library preparation techniques, and tested for genotype concordance to ensure the correct genotype. We utilized a flank-based mapping approach in the read-mapping process to avoid bias in the STR-length profiling (Gymrek et al. 2012; Fungtammasan et al. 2015). This flank-based mapping approach also removed STRs adjacent to the read termini, which were shown to exhibit high sequencing error rates (Kleinman and Majewski 2012; Pickrell et al. 2012). Finally, we included scaffolds not mapped to particular chromosomes, and removed potentially duplicated regions that were missing from the reference genome. These procedures have been demonstrated to reduce false genetic variation observed in RNA sequencing data (Peng et al. 2012), as paralogous variants could be mistaken for STR variation when an incomplete reference genome is used (Ho et al. 2011; Bass et al. 2012; Peng et al. 2012).

Several factors may still affect our RT error and RDD rate estimates. First, the RNA expression levels at the same locus vary even among cells from the same tissue. Such variation is stochastic (Elowitz et al. 2002; Ozbudak et al. 2002; Raser and O’Shea 2005; Kaufmann and van Oudenaarden 2007), and our measurement represents the mean expression level among cells in a tissue. The variation in expression level among cells in tissues could lead to improper matching between the bin size of the MLE and the expression level, which might bias our error rate estimates. One solution to alleviate this limitation in the future would be to implement single-cell RNA sequencing or the G&T-seq (simultaneous DNA- and RNA-sequencing at a single-cell level) (Saliba et al. 2014; Macaulay et al. 2015), which would enable us to estimate the number of RNA molecules expressed from a specific locus. Note that in order to use single-cell RNA sequencing data, it is necessary for the sampling from RNA to cDNA and from cDNA to sequencing reads to be modeled as multivariate hypergeometric sampling. This is necessary because there would be only a small number of actual RNA and cDNA molecules, such that the sampling process cannot be a proxy for sampling with replacement as in multinomial sampling. Despite the caveat of uncertainty in expression level, our estimations of RT error levels agree well with those estimated using barcoded RNA sequencing, which does not consider expression level information. This comparison provides an independent validation of our approach as well as points to the credibility of the estimates obtained.

Second, the MLE possibly reported a suboptimal solution if local maxima exist. However, we do not believe our estimates were distorted by this potential limitation because 1) we considered five sets of initial parameters for each bootstrap procedure of the loci and the initial parameters for the RDD and RT error rates were randomized on a log scale to accommodate error rates that have a large search space; 2) in most cases, all five of the initial parameters converged to the same solution, which suggests that the landscape of our maximum-likelihood surface may not contain many local maxima; and 3) despite the independent analysis of error rates by STR length, the estimated error rates increased exponentially with the STR length, as expected based on the known STR sequencing error and mutation patterns (Sun et al. 2012; Ross et al. 2013; Fungtammasan et al. 2015). Also, the estimation from loci with different numbers of RNA-seq reads and sequencing batches yielded similar results (fig. 2A–F).

### Conclusions and Future Directions

In this study, we provide the first model-based method to estimate the rates of RT errors and RDDs at STRs from RNA sequencing of replicated cDNA libraries. This method can be applied to existing RNA data sets with replicated cDNA libraries and a known genotype (or existing DNA-sequencing data). The merit of our approach is in that it does not require significant changes to be made to the established, general RNA-sequencing procedures. Also, our approach allows one to utilize a large number of STR loci throughout the genome, thus reducing the contextual bias due to sequencing composition around STRs. Therefore, the MLE provides a suitable alternative for estimating batched RT errors to the barcoded RNA sequencing approach (Gout et al. 2013). The currently available barcoded RNA-sequencing data are insufficient in scale to detect RDD events given the requirement that the entire STR and sufficient flanking regions need to be embedded in the reads.

Future studies should evaluate RDD error rates at STRs with more precision. Unlike RT error rates, which depend on an enzyme used for RT during library preparation, RDD rates might differ among species as they depend on species-specific biology. Moreover, both RT error and RDD rates should be evaluated for STRs others than (A)_*n*_ from a larger data set. The MLE method we developed can be used for this purpose.

A minority of repeats in our data set are heterozygous (supplementary table S1, Supplementary Material online), therefore including them would not have substantially changed our estimates, while analyzing them is computationally challenging for our model. The increased genetic polymorphism of STRs at heterozygous loci has been controversial both in terms of observations and in terms of mechanistic explanations (Amos 2016). Nevertheless, it will be interesting to analyze RDDs at heterozygous STR loci in future studies.

Another important area for future studies is the impact of RDDs on disease-causing STRs. As RDDs can modify transcripts and protein products, they could alter the phenotype and disease manifestation. Interestingly, the classification of repeat numbers for disease-causing STRs into normal, pre-mutation, and disease-causing relies on the correlation between genotype and phenotype. It is possible that, although a genotype has a non-disease repeat number, an RDD can create a disease-causing repeat in the RNA originating from the same locus. Future analyses of RDD error rates at disease-causing STRs are needed to establish the validity of such a mechanism.

## Materials and Methods

### Samples, DNA Sequencing, and Genotyping

Using the DNeasy Blood and Tissue Kit (Qiagen), we extracted genomic DNA from testis of a Bornean orangutan (*Pongo pygmaeus pygmaeus*; ID 1991-0051, Smithsonian Institute). Polymerase chain reaction (PCR)-containing and PCR-free libraries with insert size of 250–280 bp were constructed with the TruSeq DNA LT Sample Preparation Kit (Illumina) and the TruSeq DNA PCR-Free LT Sample Preparation Kit (Illumina), respectively, following the manufacturer’s protocol. The libraries were sequenced with the 150 bp × 150 bp paired-end reads on HiSeq2500 (Illumina).

The STR length arrays were profiled with STR-FM (Fungtammasan et al. 2015). Briefly, STRs with at least five mono-, three di-, three tri-, and three tetranucleotide repeats were detected in sequencing reads. We retained the reads possessing flanking regions of at least 20 bp on each side of an STR and having Phred quality score of at least 20 in the STR and their flanking regions. Flanking regions of STRs were mapped to the Sumatran orangutan (ponAbe2) reference genome with Burrows-Wheeler Aligner (BWA) (Li and Durbin 2009). We retained STRs for which both 20-bp flanking sequences mapped uniquely to the reference genome sequence. Random genomic scaffolds, that is, the ones that have not been assigned to specific chromosomes, were also used in mapping to avoid false unique mapping of some reads. STRs located closer than 10 bp to other STRs of the same class (e.g., mononucleotides) were discarded to minimize the effect of nearby STR loci on error estimation. Sequencing reads from PCR-containing and PCR-free libraries were processed separately.

For each library, the identified STR loci were genotyped using STR-FM and utilizing previously estimated sequencing error rates (Fungtammasan et al. 2015). In this step, we retained loci with a minimum of one order of magnitude difference in the probability of being the most likely homozygote versus heterozygote because such loci have a high likelihood to be genotyped correctly (Fungtammasan et al. 2015). The STR genotypes from both PCR-containing and PCR-free libraries were then compared. Discordant genotypes were removed, and the remaining genotyped loci from libraries were then combined to represent the STR length of DNA at each locus (supplementary table S1, Supplementary Material online). We limited the subsequent analysis to homozygous loci because 1) they represent the majority of our data (supplementary table S1, Supplementary Material online) and 2) heterozygous loci can display biased expression between the two alleles (Borel et al. 2015; Leung et al. 2015; Perez et al. 2015). Additionally, the use of one allele per locus simplified the model used in our MLE by reducing the number of expected STR RNA forms and their derived error forms (see “MLE Formulation” in Materials and Methods). Finally, the homologous regions between human (assembly version hg19) and orangutan (assembly version ponAbe2) genomes that had a high score in human self-alignment assembly version GRCh38 (http://hgdownload.soe.ucsc.edu/goldenPath/hg38/vsSelf/hg38.hg38.net.gz, last accessed July 16, 2016) were removed. Conversion between hg19 and GRCh38 was performed with the lift-over tool in Galaxy (Giardine et al. 2005; Blankenberg et al. 2010, 2014; Goecks et al. 2010). This removal was performed to exclude the regions in the orangutan genome that might be paralogous and might have been collapsed in the reference assembly.

### Replicated cDNA Construction, Sequencing, and Profiling

Total RNA was extracted from the same Bornean orangutan testis sample that was used for genomic DNA sequencing, with the RNeasy Mini kit protocol (Qiagen). The extracted RNA was divided into two aliquots that were utilized to generate two separate sequencing libraries (libraries 1 and 2) using the TruSeq RNA sample preparation kit (Illumina) with the stranded protocol (fig. 1). Each of the two resulting libraries was sequenced twice in two separate batches (A and B) with 150 bp × 150 bp paired-end reads on the HiSeq 2500 (Illumina).

To profile STRs in RNA, we followed the same procedure as that employed for DNA, except that we did not run the genotyping model. BWA (Li and Durbin 2009) was also employed for mapping the RNA-seq reads in our analysis to 1) minimize differences between the current procedure and the procedure used to estimate RNA sequencing errors in previous studies that also used BWA (e.g., Gout et al. 2013), 2) guard against biases that may result from applying a different algorithm for mapping RNA than for DNA, and 3) be conservative, as our preliminary results demonstrated that most STR loci that can be uniquely mapped with BWA can also be mapped with Tophat (Trapnell et al. 2009; Kim et al. 2013) and STAR (Dobin et al. 2013), whereas the opposite was not true (supplementary fig. S1, Supplementary Material online). Each RNA-seq library and each sequencing batch was analyzed separately (fig. 1). As a result, we conducted identical analyses on four different library–batch combinations.

### MLE Formulation

We formulated the MLE to infer four parameters—RDD rate, RDD expansion probability, RT error rate, and RT expansion probability—that maximize the probability of observed data (maximum-likelihood estimation of the parameters) in STRs obtained from RNA-seq. The model includes an expansion probability for RDD, *p*_RDD_, and the contraction probability can be computed as 1 – *p*_RDD_. The same is true for an expansion probability for RT errors, *p*_RT_. The model used in the MLE is based on the following key assumptions:

1. All loci are independent.
2. The error rates and the expansion probabilities for both RT errors and RDDs are identical for all DNA loci with the same STR motif and repeat number.
3. Both RT errors and RDDs follow the stepwise mutation model (Kimura and Ohta 1978; Valdes et al. 1993; Di Rienzo et al. 1998; Sainudiin et al. 2004) that only allows expansion or contraction by one repeat unit after a single round of each process (i.e., transcription or RT). Thus, starting from DNA (e.g., (AG)_6_), there are three possible STR forms for RNA (e.g., (AG)_5_, (AG)_6_, and (AG)_7_), and five possible STR forms for cDNA (e.g., (AG)_4_, (AG)_5_, (AG)_6_, (AG)_7_, and (AG)_8_). We denote the number of possible STR forms at a given stage (RNA or cDNA) as *K*.
4. The model uses a fixed bin size (denoted by *M*), which represents the number of sampled RNA or cDNA molecules after transcription or RT, respectively. This finite bin size *M* permits alterations in the expected distribution of STR forms in a given stage (RNA or cDNA) by conditioning on the number of STRs of a given form passed on from the previous stage. For example, suppose that at the RNA stage the relative proportions of STR forms for four, five, and six repeats are 0.1, 0.5, and 0.4, respectively. Based on the previous point, five possible cDNA forms are expected—those with three, four, five, six, and seven repeats. If we sample only a small number *M* of STRs to be passed from the RNA to the cDNA stage, then it is likely that the STR form with three repeats will not be represented in the cDNA stage. However, if *M* is sufficiently large, then the probability of observing all possible forms at the cDNA stage is high. This sampling permits different cDNA libraries for the same RNA sample to be correlated, as the STR forms observed in these libraries are conditional on the RNA STR forms that they share, and allows us to take advantage of the structure of our experimental design (fig. 1). We use this bin size to generate all possible compositions of STR length for RNA and cDNA, depending on expression level proxied by the number of RNA-seq reads (see below). For example, for DNA with an STR of (AG)_6_ and the bin size of 2, there are six possible compositions of RNA forms (i.e., (AG)_5_(AG)_5_, (AG)_5_(AG)_6_, (AG)_5_(AG)_7_, (AG)_6_(AG)_6_, (AG)_6_(AG)_7_, and (AG)_7_(AG)_7_) and 15 possible compositions of cDNA forms (i.e., (AG)_4_(AG)_4_, (AG)_4_(AG)_5_, … , and (AG)_8_(AG)_8_). Considering all possible compositions allows us to calculate the probability of changes from DNA to RNA, and from RNA to cDNA, which permits the derivation of the distribution of STR forms at the RNA and the cDNA stages.With this formulation, the likelihood function at locus *j* can be represented as
  $$L\left(\text{θ; data }j\right)\;=\;\sum_{c1}\sum_{c2}P\left(\text{data }j|c1,\;c2\right)\;\sum_{r}P\;\left(c1,\;c2|r,\theta \right)P(r|\theta ),$$

where θ is a vector of model parameters; *c*1 and *c*2 are vectors of the numbers of STRs at each STR form in cDNA libraries 1 and 2, respectively; and *r* is a vector of the numbers of STRs at the RNA stage. The log likelihood of the data at all *L* loci is then

$$\ell (\text{θ; data 1, 2, }\ldots \text{, }L)\;=\;\sum_{j\;=\;1}^{L}\log[L(\text{θ; data }j)].$$

Note that for a bin size of *M* at a stage with *K* possible STR forms, the number of compositions is *M *+* K* − 1 choose *K* − 1, which grows quickly as the bin size *M* increases. See supplementary text S1, Supplementary Material online, for the derivation of our MLE. Note that the bin size incorporates expression level into the model. In practical terms, and assuming RNA sequencing was performed at high depth to capture the vast majority of unique transcripts, expression level is proxied by the number of RNA-seq reads for each locus.

We calculated the probability that the observed data are generated from all possible distributions of RNA and cDNA STR lengths for a given number of sampled molecules *M* under the stepwise mutation model (assumptions 3 and 4), to ensure that the estimation is not distorted by an incorrect inference of RNA and cDNA STR profiles. In the transition from cDNA to sequencing reads, we incorporated the sequencing error rates estimated by Fungtammasan et al. (2015).

The MLE was implemented in R (R Development Core Team). We chose the L-BFGS-B (Limited-memory Broyden–Fletcher–Goldfarb–Shanno with box constraints) method (Byrd et al. 1995; Malouf 2002) from the “optim” function for parameter searching. The box constraints (parameter limits) were set from 10^−^^9^ to 0.5 for the RT error and RDD rates, and from 0 to 1 for the expansion probabilities. We used the lower bound of 10^−^^9^ as it is several orders of magnitude lower than known STR germ-line mutation rates (10^−^^5^–10^−^^2^; Sun et al. 2012; Fungtammasan et al. 2015). The upper bound of 0.5 assumes that half of the reads are erroneous. For the expansion probability, a value of 0 indicates all contractions, 0.5 indicates an equal ratio between expansions and contractions, and 1 indicates all expansions.

### Lumping MLE

Due to the computationally intensive nature of the algorithm when bin size *M* is large, we also considered a reduced form of the model that lumps the two cDNA forms with smallest repeat number into one class and the two forms with the largest repeat number into another class. That is, in our original model there are five cDNA forms with repeat numbers *D* − *2, D* − *1, D, D + 1*, and *D + 2*, where *D* is the DNA STR length. In this modified approach, we lump forms with length *D* − *2* and *D* − *1* into a single form *(D* − *1)lump* and lump forms *D + 1* and *D + 2* into a single form *(D + 1)lump*. This formulation reduces the complexity of the calculation as we now have *K = 3* forms instead of *K = 5* forms at the cDNA stage, and this substantially reduces the number of compositions needed to be evaluated from *M + 5* − *1* choose 5 − 1 to *M + 3* − *1* choose 3 − 1, thereby permitting consideration of larger bin sizes for a fixed amount of computing time. The probability of state *(D* − *1)lump* is the sum of the probabilities of states *D* − *2* and *D* − *1*, and the probability of state *(D + 1)lump* is the sum of the probabilities of states *D + 1* and *D + 2*. As an example, if the genotype is (A)_8_, then based on the stepwise mutation model there are three possible forms of RNA ((A)_7_, (A)_8_, (A)_9_) and five possible forms of cDNA ((A)_6_, (A)_7_, (A)_8_, (A)_9_, (A)_10_). We lump the probabilities of (A)_6_ and (A)_7_ and those of (A)_9_ with (A)_10_ to reduce the complexity of the calculation. We will refer to this algorithm as “lumping MLE.” Its full description can be found in supplementary text S2, Supplementary Material online. The full and lumping MLE were implemented in R and the resulting software, STR-RNA-MLE, can be downloaded from https://github.com/Arkarachai/str-rna-mle.

### MLE Method Evaluation

To test the ability of our method to estimate its four parameters, we performed simulations to generate random STR length profiles based on fixed RT error and RDD rates (0.01 or 0.05) and expansion probabilities (0.3, 0.7, or 0.8), the number of studied loci (10, 100, 1,000, or 10,000), two replicated cDNA libraries, and the number of RNA and cDNA molecules (ranging from 2 to 17 molecules). We then employed our MLE to infer the parameters for each simulation set using bin sizes of 2, 3, and 5. We generated 100 replicate data sets for a given parameter set, and estimated the parameters for each replicate. The 95% confidence interval for each estimated parameter was calculated from the average of the second and third lowest inferred values to obtain the lower bound, and the average of the second and third highest inferred values to obtain the upper bound, from a set of 100 replicates. We also tested the lumping MLE by comparing the estimated parameters from the data with the number of RNA and cDNA molecules set at 6 and 10, RT error and RDD rates set at 0.1, expansion probabilities of RT errors and RDDs of 0.8, 1,000 loci, and two cDNA libraries using both our standard MLE model and lumping MLE model at a bin size equal to 5.

### Estimation of RDD and RT Errors Using the MLE from the Orangutan Data

For each batch of RNA sequencing, the RNA profiling data of replicated libraries were paired with the DNA genotypes at the same loci. Each batch of replicated sequencing data was analyzed separately. We selected subsets of data with an appropriate number of RNA-seq reads to analyze specific bin sizes. Initially, we chose a bin size of 2 and analyzed a subset of data that had a mean number of RNA-seq reads of three to five reads per locus, requiring a minimum of two reads. The loci within this range of RNA-seq reads were chosen because 1) 65% of the STR-containing loci in our data set have low expression level (with less than five reads per locus; supplementary fig. S2, Supplementary Material online) and 2) their estimated rates of RT errors and RDDs are less than 2-fold different from the expected values based on our simulations (supplementary figs. S3 and S4, Supplementary Material online). To ensure that our estimated rates are valid for the loci with higher expression levels, we used the lumping MLE model to analyze 1) a subset of data with 6–16 RNA-seq reads per locus, using a bin size of 5, and 2) a subset of data with 49–102 RNA-seq reads per locus, using a bin size of 40. For each sequencing batch, each STR length, and each binning of the data, we generated 100 bootstrap replicates in which the loci had the same DNA length and STR length profiles as in our RNA sequencing data from the two replicated cDNA libraries. We analyzed each bootstrap replicate with the MLE starting with five random initial sets of the four parameters of interest, and chose the parameter estimates with the highest likelihood. We started with five initial parameter sets to avoid hitting local maxima. We then calculated the 95% confidence interval for each of the four model parameters by taking the average of the second and third lowest inferred values as the lowered bound, and the average of the second and third highest values as the upper bound, with parameters estimated at each of 100 bootstrap replicates.

### Analysis of the Barcoded mRNA Sequencing Data

To verify the RT error and RDD rates estimated with the MLE, we evaluated them using the publicly available barcoded RNA sequencing data (Gout et al. 2013) to which we applied the same stringent filtering parameters (see “Replicated cDNA Construction, Sequencing, and Profiling” in Materials and Methods). The published RNA sequencing data are derived from three different strains of *Caenorhabditis elegans* (Gout et al. 2013)*.* In this data set, the RNA extracted from each strain was barcoded and then reverse transcribed sequentially three times, and each product of RT was sequenced separately. Based on the barcodes, we traced all sequencing reads that belonged to the same original RNA molecule (referred to as “family”) based on the shared barcode and shared starting genomic mapping coordinate (supplementary fig. S5, Supplementary Material online). We mapped flanking regions of the STR-containing sequencing reads to the *C. elegans* genome assembly version Ce10 using BWA (Li and Durbin 2009), and applied a modified STR-FM pipeline (Fungtammasan et al. 2015). To reduce sequencing errors, at least two STR-containing reads from the same family were used to infer one STR-containing molecule at the cDNA step for each library (note that if two reads mapping to the same locus had STR lengths of 10 and 11, we did not infer cDNA state for this locus in this library). Ideally, these two STR-containing reads should come from overlapping paired-end reads (Gout et al. 2013). However, due to our requirement that STR and 20 bp of their flanking regions upstream and downstream must be located within the reads, we found only six pairs of reads that came from overlapping paired-end reads. Therefore, to infer STR length at a cDNA molecule, instead of using overlapping paired-end reads, we considered all reads from the same family in a library, even though some of them might have constituted PCR duplicates. Next, to infer STR lengths at RNA molecules and RT errors, we utilized cDNA STRs from the same family present in at least two cDNA libraries. Finally, to infer RDDs, we collected all inferred RNA molecules that mapped to the same STR locus.

The rates of RT errors of RDDs were calculated from the proportion of reads with incongruent STR length per locus. For example, if in a cDNA library STR reads with lengths of 10, 10, and 11 bp belonged to the same family, then we inferred the consensus RNA to have an STR length of 10 bp, two cDNA reads with no RT errors, and one cDNA read with a 1-bp expansion RT error. If we observed only two cDNA reads that differed from each other, then we used the consensus length (the unambiguous majority length of STR reads mapping to that locus regardless of the family or library), to polarize the direction of an error. For example, if two cDNA reads in a certain family had an STR with lengths of 10 and 11 bp at the same locus, and the most common cDNA STR length for all the families at this STR locus was 10 bp, then we inferred that the STR of 11 bp was erroneous. Our error estimation did not include any cDNA families or RNA molecules for which we could infer only one cDNA molecule, or one RNA molecule, at a certain locus, as errors could not be inferred in such cases.

As an alternative method of comparison, the RNA sequencing STR length profile of *C. elegans* (Gout et al. 2013) was also used to estimate the RDD and RT error rates inferred using MLE without utilizing the barcode information. For each of the three *C. elegans* strains, the two (out of three) replicated cDNA libraries with the highest sequencing depths were chosen, and the data were processed exactly as for the orangutan data above (with *M *= 2). To infer cDNA molecules, we employed the sequencing error rates from Fungtammasan et al. (2015) instead of using the information from barcoded RNA sequencing reads.

## Supplementary Material

Supplementary texts S1–S3, figures S1–S12, and tables S1–S10 are available at *Molecular Biology and Evolution* online (http://www.mbe.oxfordjournals.org/).

Supplementary Data
