# Supplementary material for: Reverse Transcription Errors and RNA–DNA Differences at Short Tandem Repeats
Source: Mol Biol Evol. 2016 Jul 12;33(10):2744–58. doi: 10.1093/molbev/msw139 (PMC5026258; doi:10.1093/molbev/msw139)
Supplement: Supplementary Data [file supp_33_10_2744__index.html]

Reverse Transcription Errors and RNA–DNA Differences at Short Tandem Repeats — Supplementary Data 

# Reverse Transcription Errors and RNA–DNA Differences at Short Tandem Repeats

## Supplementary Data

files

- Supplementary Data - zip file
