# Supplementary material for: Reverse Transcription Errors and RNA–DNA Differences at Short Tandem Repeats
Source: Mol Biol Evol. 2016 Jul 12;33(10):2744–58. doi: 10.1093/molbev/msw139 (PMC5026258; doi:10.1093/molbev/msw139)
Supplement: Supplementary Data [file supp_msw139_suppl_data.zip › Text3_ChaiMicrosat_Multinomial_4lib.pdf]

Maximum likelihood estimator with four libraries

Suppose that the initial DNA length is D and that the vector of parameters is

$$\boldsymbol{\theta} = (\epsilon_{\text{RDD}}, \epsilon_{\text{RT}}, p_{\text{RDD}}, p_{\text{RT}}).$$

Let

$$\mathbf{r} = (r_{D-1}, r_D, r_{D+1})$$

be the vector of counts for microsatellites at the RNA step with lengths $D-1$, D, and $D+1$ such that $\sum_{k=D-1}^{D+1} r_k = M$, with M an integer that is at minimum the sequencing depth (large values of M are likely better). Let

$$\mathbf{c}^{(1)} = (c_{D-2}^{(1)}, c_{D-1}^{(1)}, c_D^{(1)}, c_{D+1}^{(1)}, c_{D+2}^{(1)})$$
$$\mathbf{c}^{(2)} = (c_{D-2}^{(2)}, c_{D-1}^{(2)}, c_D^{(2)}, c_{D+1}^{(2)}, c_{D+2}^{(2)})$$

be the vectors of counts for microsatellites at the CDNA1 step and CDNA2 step, respectively, such that $\sum_{k=D-2}^{D+2} c_k^{(i)} = M$ for CDNA step i. Let

$$\mathbf{y}^{(1)} = (y_1^{(1)}, y_2^{(1)}, \dots, y_N^{(1)})$$
$$\mathbf{y}^{(2)} = (y_1^{(2)}, y_2^{(2)}, \dots, y_N^{(2)})$$
$$\cdots$$
$$\mathbf{y}^{(4)} = (y_1^{(4)}, y_2^{(4)}, \dots, y_N^{(4)})$$

be the vectors of observed microsatellite counts in each of the 4 sequences, respectively.

Define the collection of matrix of counts $\mathbf{Y} = (\mathbf{y}^{(1)}, \dots, \mathbf{y}^{(4)})$. We want to calculate $\mathbb{P}[\mathbf{Y} \,|\, \boldsymbol{\theta}]$ so that we can compute the likelihood $\mathcal{L}(\boldsymbol{\theta}; \mathbf{Y}) = \mathbb{P}[\mathbf{Y} \,|\, \boldsymbol{\theta}]$. Using the hierarchical structure of the experimental design, we have that

$$\begin{aligned}
\mathbb{P}[\mathbf{Y} \,|\, \boldsymbol{\theta}] &= \sum_{\mathbf{c}^{(1)}} \sum_{\mathbf{c}^{(2)}} \mathbb{P}[\mathbf{Y} \,|\mathbf{c}^{(1)}, \mathbf{c}^{(2)}] \mathbb{P}[\mathbf{c}^{(1)}, \mathbf{c}^{(2)} \,|\, \boldsymbol{\theta}] \\
&= \sum_{\mathbf{c}^{(1)}} \sum_{\mathbf{c}^{(2)}} \left(\prod_{i=1}^{2} \mathbb{P}[\mathbf{y}^{(i)} \,|\, \mathbf{c}^{(1)}] \right) \left(\prod_{i=3}^{4} \mathbb{P}[\mathbf{y}^{(i)} \,|\, \mathbf{c}^{(2)}] \right) \mathbb{P}[\mathbf{c}^{(1)}, \mathbf{c}^{(2)} \,|\, \boldsymbol{\theta}] \\
&= \sum_{\mathbf{c}^{(1)}} \left(\prod_{i=1}^{2} \mathbb{P}[\mathbf{y}^{(i)} \,|\, \mathbf{c}^{(1)}] \right) \sum_{\mathbf{c}^{(2)}} \left(\prod_{i=3}^{4} \mathbb{P}[\mathbf{y}^{(i)} \,|\, \mathbf{c}^{(2)}] \right) \mathbb{P}[\mathbf{c}^{(1)}, \mathbf{c}^{(2)} \,|\, \boldsymbol{\theta}] \\
&= \sum_{c_{D-2}^{(1)}=0}^{M} \sum_{c_{D-1}^{(1)}=0}^{M-c_{D-2}^{(1)}} \sum_{c_{D}^{(1)}=0}^{M-c_{D-2}^{(1)}-c_{D-1}^{(1)}} \sum_{c_{D+1}^{(1)}=0}^{M-c_{D-2}^{(1)}-c_{D-1}^{(1)}-c_{D}^{(1)}} \left[\left(\prod_{i=1}^{2} \mathbb{P}[\mathbf{y}^{(i)} \,|\, \mathbf{c}^{(1)}] \right) \right. \\
&\qquad \times \left. \sum_{c_{D-2}^{(2)}=0}^{M} \sum_{c_{D-1}^{(2)}=0}^{M-c_{D-2}^{(2)}} \sum_{c_{D}^{(2)}=0}^{M-c_{D-2}^{(2)}-c_{D-1}^{(2)}} \sum_{c_{D+1}^{(2)}=0}^{M-c_{D-2}^{(2)}-c_{D-1}^{(2)}-c_{D}^{(2)}} \left(\prod_{i=3}^{4} \mathbb{P}[\mathbf{y}^{(i)} \,|\, \mathbf{c}^{(2)}] \right) \mathbb{P}[\mathbf{c}^{(1)}, \mathbf{c}^{(2)} \,|\, \boldsymbol{\theta}] \right] \\
&= \sum_{c_{D-2}^{(1)}=0}^{M} \sum_{c_{D-1}^{(1)}=0}^{M-c_{D-2}^{(1)}} \sum_{c_{D}^{(1)}=0}^{M-c_{D-2}^{(1)}-c_{D-1}^{(1)}} \sum_{c_{D+1}^{(1)}=0}^{M-c_{D-2}^{(1)}-c_{D-1}^{(1)}-c_{D}^{(1)}} \left[\left(\prod_{i=1}^{2} P(\mathbf{y}^{(i)} \,;\, \mathbf{p}^{\text{Seq},1}) \right) \right. \\
&\qquad \times \left. \sum_{c_{D-2}^{(2)}=0}^{M} \sum_{c_{D-1}^{(2)}=0}^{M-c_{D-2}^{(2)}} \sum_{c_{D}^{(2)}=0}^{M-c_{D-2}^{(2)}-c_{D-1}^{(2)}} \sum_{c_{D+1}^{(2)}=0}^{M-c_{D-2}^{(2)}-c_{D-1}^{(2)}-c_{D}^{(2)}} \left(\prod_{i=3}^{4} P(\mathbf{y}^{(i)} \,;\, \mathbf{p}^{\text{Seq},2}) \right) \mathbb{P}[\mathbf{c}^{(1)}, \mathbf{c}^{(2)} \,|\, \boldsymbol{\theta}] \right],
\end{aligned}$$

where $P(\mathbf{y}^{(i)}; \mathbf{p}^{\text{Seq},j})$ is the probability mass function of a multinomial distribution with counts $\mathbf{y}^{(i)}$ and parameterized by probabilities $\mathbf{p}^{\text{Seq},j}$, where the kth term ($k = 1, 2, \ldots, N$) of $\mathbf{p}^{\text{Seq},j}$ is

$$p_k^{\text{Seq},j} = \sum_{\ell=D-2}^{D+2} \frac{c_\ell^{(j)}}{M} P_{\ell k}^{\text{Chai}},$$

where $P_{\ell k}^{\text{Chai}}$ is the probability that microsatellite A_ℓ will become A_k due to sequencing error (from Chai's paper). Therefore

$$P(\mathbf{y}^{(i)}; \mathbf{p}^{\text{Seq},j}) = \binom{||\mathbf{y}^{(i)}||_1}{\mathbf{y}^{(i)}} \prod_{k=1}^{N} (p_k^{\text{Seq},j})^{y_k^{(i)}}.$$

Next, we find

$$\begin{aligned}
\mathbb{P}[\mathbf{c}^{(1)}, \mathbf{c}^{(2)} \mid \boldsymbol{\theta}] &= \sum_{\mathbf{r}} \mathbb{P}[\mathbf{c}^{(1)}, \mathbf{c}^{(2)} \mid \mathbf{r}, \boldsymbol{\theta}] \mathbb{P}[\mathbf{r} \mid \boldsymbol{\theta}] \\
&= \sum_{\mathbf{r}} \mathbb{P}[\mathbf{c}^{(1)} \mid \mathbf{r}, \boldsymbol{\theta}] \mathbb{P}[\mathbf{c}^{(2)} \mid \mathbf{r}, \boldsymbol{\theta}] \mathbb{P}[\mathbf{r} \mid \boldsymbol{\theta}] \\
&= \sum_{r_{D-1}=0}^{M} \sum_{r_D=0}^{M-r_{D-1}} \mathbb{P}[\mathbf{c}^{(1)} \mid \mathbf{r}, \boldsymbol{\theta}] \mathbb{P}[\mathbf{c}^{(2)} \mid \mathbf{r}, \boldsymbol{\theta}] \mathbb{P}[\mathbf{r} \mid \boldsymbol{\theta}] \\
&= \sum_{r_{D-1}=0}^{M} \sum_{r_D=0}^{M-r_{D-1}} P(\mathbf{c}^{(1)}\,;\, \mathbf{p}^{\text{CDNA}}) P(\mathbf{c}^{(2)}\,;\, \mathbf{p}^{\text{CDNA}}) \mathbb{P}[\mathbf{r} \mid \boldsymbol{\theta}],
\end{aligned}$$

where $P(\mathbf{c}^{(i)}\,;\, \mathbf{p}^{\text{CDNA}})$ is the probability mass function of a multinomial distribution with counts $\mathbf{c}^{(i)}$ and parameterized by probabilities $\mathbf{p}^{\text{CDNA}}$, where the kth term ($k = D-2, D-1, \ldots, D+2$) of $\mathbf{p}^{\text{CDNA}}$ is

$$p_k^{\text{CDNA}} = \sum_{j=D-1}^{D+1} \frac{r_j}{M} P_{jk}^{(2)},$$

where $P_{jk}^{(2)}$ is the probability that microsatellite A_j will become A_k due to the CDNA step, and is defined as

$$P_{jk}^{(2)} = \begin{cases} \epsilon_{\text{RT}}(1 - p_{\text{RT}}) & \text{if } k = j - 1 \\ 1 - \epsilon_{\text{RT}} & \text{if } k = j \\ \epsilon_{\text{RT}} p_{\text{RT}} & \text{if } k = j + 1 \\ 0 & \text{otherwise} \end{cases}$$

Therefore

$$P(\mathbf{c}^{(i)}\,;\, \mathbf{p}^{\text{CDNA}}) = \binom{M}{c_{D-2}^{(i)} \cdots c_{D+2}^{(i)}} \prod_{k=D-2}^{D+2} (p_k^{\text{CDNA}})^{c_k^{(i)}}.$$

Finally, we have

$$\mathbb{P}[\mathbf{r} \mid \boldsymbol{\theta}] = P(\mathbf{r}\,;\, \mathbf{p}^{\text{RNA}})$$

where $P(\mathbf{r}\,;\, \mathbf{p}^{\text{RNA}})$ is the probability mass function of a multinomial distribution with counts $\mathbf{r}$ and parameterized by probabilities $\mathbf{p}^{\text{RNA}}$, where the kth term ($k = D-1, D, D+1$) of $\mathbf{p}^{\text{RNA}}$ is

$$p_k^{\text{RNA}} = P_{Dk}^{(1)},$$

where $P_{Dk}^{(1)}$ is the probability that microsatellite A_D will become A_k due to the RNA step, and is defined as

$$P_{Dk}^{(1)} = \begin{cases} \epsilon_{\text{RDD}}(1 - p_{\text{RDD}}) & \text{if } k = D - 1 \\ 1 - \epsilon_{\text{RDD}} & \text{if } k = D \\ \epsilon_{\text{RDD}} p_{\text{RDD}} & \text{if } k = D + 1 \\ 0 & \text{otherwise} \end{cases}$$

Therefore

$$P(\mathbf{r}\,;\, \mathbf{p}^{\text{RNA}}) = \binom{M}{r_{D-1}\, r_D\, r_{D+1}} \prod_{k=D-1}^{D+1} (p_k^{\text{RNA}})^{r_k^{(i)}}.$$

Now, suppose that we have L independent loci, for which we have the matrix of STR profiles (counts) $\mathbf{Y}_j$ at locus j, $j = 1, 2, \ldots, L$. The log likelihood of the parameters $\boldsymbol{\theta}$ taken across all loci is

$$\ell(\boldsymbol{\theta}; \mathbf{Y}_1, \mathbf{Y}_2, \ldots, \mathbf{Y}_L) = \sum_{j=1}^{L} \log[\mathcal{L}(\boldsymbol{\theta}; \mathbf{Y}_j)].$$

We identify the set of parameters $\boldsymbol{\theta}$ that maximizes this log likelihood function.
