# Supplementary material for: Reverse Transcription Errors and RNA–DNA Differences at Short Tandem Repeats
Source: Mol Biol Evol. 2016 Jul 12;33(10):2744–58. doi: 10.1093/molbev/msw139 (PMC5026258; doi:10.1093/molbev/msw139)

SUPPLEMENTAL TABLES

Table S1. The number of successfully genotyped STR loci in the orangutan DNA sample sequenced using two different library construction protocols.

	**Number of loci**
**PCR-containing library sequencing***	8,858,973 (8,823,257 homozygotes and, 35,716 heterozygotes)
**PCR-free library sequencing***	699,215 (696,830 homozygotes and 2,385 heterozygotes)
**Loci shared between the two libraries**	550,482
**Loci with concordant genotypes between the two libraries**	549,716 (99.86%)
**Merged loci (loci that were sequenced in either the PCR-containing or the PCR-free library plus shared loci with concordant genotypes)**	9,007,706 (99.55% homozygotes and 0.45% heterozygotes)
**Merged homozygous loci**	8,967,575
**Merged homozygous loci after discarding orthologs to human duplicated regions**	8,738,460

*Note that the PCR-containing library was sequenced at 267 million reads, while the PCR-free library sequencing library was sequenced at 73 million reads.

Table S2. The number of STR loci detected in at least one library in each batch of the orangutan RNA-seq data as a function of repeat motif.

Repeated motif	Sequencing batch A	Sequencing batch B
A	72,102	72,370
C	15,681	15,869
AG	22,669	23,229
AC	15,534	15,741
AT	8,446	8,493
CG	197	189
All trinucleotide STRs	5,394	5,261
All tetranucleotide STR	976	961

Table S3. The estimated parameters from full MLE model and lumping MLE with the bin size of 5. Parameters used: RDD rate = 0.01, p_{RDD} = 0.8, RT error rate = 0.01, p_{RT} = 0.8; 1,000 loci.

**Number of RNA and cDNA molecules**	**Expected parameters**	**Full MLE estimation**		**Lumping MLE estimation**	
		**Median**	**95% C.I.**	**Median**	**95% C.I.**
6	RDD rate	0.0140	[0.0076, 0.0219]	0.0142	[0.0075, 0.0223]
	RDD expansion probability	0.8076	[0.6343, 0.9672]	0.8112	[0.6334, 0.9655]
	RT error rate	0.0067	[0.0029, 0.0100]	0.0064	[0.0018, 0.0101]
	RT expansion probability	0.6135	[0.2129, 0.8765]	0.5940	[0.1157, 0.9430]
10 (optimal)	RDD rate	0.0104	[0.0064, 0.0146]	0.0103	[0.0063, 0.0147]
	RDD expansion probability	0.8077	[0.6388, 1.0]	0.8118	[0.6420, 1.0]
	RT error rate	0.0128	[0.0091, 0.0160]	0.0128	[0.0091, 0.0160]
	RT expansion probability	0.6809	[0.5707, 0.7944]	0.6811	[0.5760, 0.7954]

Table S4. RDD rates, RDD expansion probability, RT error rates, and RT expansion probability of the $(A)_n$ motif estimated from sequencing (A) batch A, and (B) batch B, using the bin size of two and three to five RNA-seq reads per locus.

A

STR length (n)	Number of loci	Parameter	Point estimation	Bootstrap estimation		
				Median	Lower bound of 95% C.I.	Upper bound of 95% C.I.
5	8975	RDD rate	< 1.0e-9	< 1.0e-9	< 1.0e-9	< 1.0e-9
		RDD expansion probability	-	-	-	-
		RT error rate	2.79e-4	2.81e-4	1.15e-4	4.49e-4
		RT expansion probability	0.99	0.99	0.99	0.99
6	2388	RDD rate	1.87e-3	1.86e-3	2.45e-4	3.38e-3
		RDD expansion probability	1	1	1	1
		RT error rate	8.85e-3	8.94e-3	7.09e-3	1.12e-2
		RT expansion probability	0.81	0.82	0.72	0.90
7	878	RDD rate	< 1.0e-9	< 1.0e-9	< 1.0e-9	2.28e-3
		RDD expansion probability	-	0.68	0.07	1
		RT error rate	1.85e-2	1.86e-2	1.44e-2	2.27e-2
		RT expansion probability	0.73	0.73	0.61	0.85
8	314	RDD rate	3.57e-3	4.79e-3	< 1.0e-9	1.48e-2
		RDD expansion probability	0.99	0.85	0	1
		RT error rate	3.53e-2	3.38e-2	2.32e-2	4.49e-2
		RT expansion probability	0.62	0.62	0.42	0.80

9	145	RDD rate	8.94e-3	8.52e-3	< 1.0e-9	2.28e-2
		RDD expansion probability	0	0	0	0.89
		RT error rate	5.85e-2	5.66e-2	3.55e-2	8.21e-2
		RT expansion probability	0.65	0.64	0.49	0.84
10	45	RDD rate	< 1.0e-9	< 1.0e-9	< 1.0e-9	6.55e-2
		RDD expansion probability	-	0.43	0	0.99
		RT error rate	9.71e-2	9.53e-2	3.82e-2	1.44e-1
		RT expansion probability	0.61	0.64	0.32	0.99

B

STR length (n)	Number of loci	Parameter	Point estimation	Bootstrap estimation		
				Median	Lower bound of 95% C.I.	Upper bound of 95% C.I.
5	7567	RDD rates	< 1.0e-9	6.91e-5	< 1.0e-9	2.76e-4
		RDD expansion probability	-	0.99	0.15	1
		RT error rates	2.14e-4	2.46e-4	4.17e-5	4.25e-4
		RT expansion probability	0.99	0.99	0.99	0.99
6	2022	RDD rates	5.13e-4	3.47e-4	< 1.0e-9	2.01e-3
		RDD expansion probability	0.97	1	0.25	1
		RT error rates	1.39e-2	1.39e-2	1.11e-2	1.68e-2
		RT expansion probability	0.93	0.94	0.88	0.99
7	779	RDD rates	2.59e-3	2.44e-3	< 1.0e-9	7.59e-3
		RDD expansion probability	0.35	0.28	0	1
		RT error rates	1.76e-2	1.76e-2	1.27e-2	2.34e-2
		RT expansion probability	0.67	0.67	0.52	0.83
8	268	RDD rates	2.68e-3	3.61e-3	< 1.0e-9	1.72e-2
		RDD expansion probability	0	0.23	0	1
		RT error rates	3.17e-2	3.15e-2	1.67e-2	4.42e-2
		RT expansion probability	0.81	0.81	0.60	0.99
9	108	RDD rates	< 1.0e-9	< 1.0e-9	< 1.0e-9	< 1.0e-9
		RDD expansion probability	-	-	-	-
		RT error	7.73e-2	7.49e-2	4.69e-2	1.07e-1

		rates				
		RT expansion probability	0.57	0.58	0.36	0.79
10	53	RDD rates	1.15e-2	1.29e-2	< 1.0e-9	8.22e-2
		RDD expansion probability	0	0.40	0	1
		RT error rates	8.88e-2	8.12e-2	2.64e-2	1.37e-1
		RT expansion probability	0.75	0.77	0.23	0.99

Table S5. The number of loci that were included in the lumping MLE estimation for the orangutan data.

**Repeat number $(A)_n$**	**Bin size = 5; 6-16 RNA-seq reads per locus**		**Bin size = 40; 49-102 RNA-seq reads per locus**	
	**Batch A**	**Batch B**	**Batch A**	**Batch B**
**5**	7494	6312	854	699
**6**	1888	1647	242	200
**7**	766	629	111	100
**8**	247	180	19	22
**9**	121	100	15	12
**10**	43	30	4	3

Table S6. The number of loci for each repeat number and the number of RNA-seq reads per locus.

Repeat number	Number of RNA-seq reads	AG and AC repeat		Trinucleotide repeat		Tetranucleotide repeat	
		Batch A	Batch B	Batch A	Batch B	Batch A	Batch B
**3**	**3-5**	4597	4017	567	456	52	62
	**6-16**	4236	4000	526	506	59	56
	**49-102**	509	532	71	61	10	12
**4**	**3-5**	477	349	24	26	6	2
	**6-16**	421	383	40	29	4	4
	**49-102**	57	56	3	4	0	0
**5**	**3-5**	68	46	4	3	0	0
	**6-16**	56	57	8	7	1	1
	**49-102**	6	7	0	0	0	0

Table S7. Estimated RT error rates and RT expansion probability of $(AG)_n$ and $(AC)_n$ repeats for batch A and B. In each cell, the number outside the brackets is the point estimation, while the numbers inside the brackets are lower and upper bounds of the 95% confidence intervals. NA cells are repeat number that with no found deviant STR repeat variants.

Bin size	Number of RNA-seq reads	Repeat number (n)	RT expansion rates		RT expansion probabilities	
			Batch A	Batch B	Batch A	Batch B
2	3-5	3	NA	NA	NA	NA
		4	5.84e-4 [<1.0e-9, 1.76e-3]	7.08e-4 [<10e-9, 3.19e-3]	0 [0, 0.98]	0 [0, 0.99]
		5	NA	1.77e-3 [<10e-9, 1.75e-2]	NA	0 [0, 0.93]
5	6-16	3	NA	NA	NA	NA
		4	8.59e-4 [<10e-9, 2.28e-3]	3.24e-4 [<10e-9, 1.13e-3]	0 [0, 0.95]	0 [0, 0.98]
		5	NA	1.60e-3 [<10e-9, 7.01e-3]	NA	0 [0, 0.95]

Table S8. The number of reads, molecules, and error events observed in the barcoded RNA-seq data.

	Wild-type strain	VC1305 strain	RB886 strain	Total
**Total STR-containing reads**	2,858,496	2,873,936	3,342,258	9,074,690
**Inferred cDNA molecule**	366,979	270,669	312,178	949,826
**Inferred RNA molecule**	3,702	1,374	2,846	7,922
**RT error event**	8	0	4	12
**RDD event**	0	0	0	0

Table S9. The number of STR-containing loci in inferred STR molecules from the barcoded RNA sequencing data as a function of repeat length.

Mononucleotide STRs

Repeat length (bp)	A	C
	Number of loci	Number of loci
**5**	3549	242
**6**	641	14
**7**	92	0
**8**	23	0
**9**	28	4
**10**	2	0
**11**	1	0
**Total**	4336	260

Dinucleotide STRs

Repeat length (bp)	AT	AC	AG	CG
**6**	27	525	478	42
**7**	46	53	148	4
**8**	3	13	9	0
**9**	1	1	10	1
**10**	1	1	0	0
**11**	0	3	0	0
**12**	0	1	0	0

**13**	0	7	0	0
**16**	0	1	0	0
**17**	0	1	0	0
**Total**	78	606	645	47

Tri- and tetranucleotide STRs

**Repeat length (bp)**	**Trinucleotide STR**	**Tetranucleotide STR**
**9**	745	0
**10**	529	0
**11**	655	0
**12**	6	0
**13**	5	0
**14**	5	2
**16**	3	0
**Total**	1948	2

Table S10. RT error profiles of mononucleotide STRs with motif $(A)_n$ from the barcoded RNA sequencing. The original lengths were inferred from the consensus STRs in all reads mapped to the same location. The cDNA families present in only one sequencing library were not included, as these could not be used to infer errors.

STR length (bp)		cDNA count				Percentage	Error rate	Expansion probability
Original length	Observed length	Wild-type	VC1305	RB886	Total			
5	5	4233	853	2435	7521	99.987%	1.33E-04	1
5	6	1	0	0	1	0.013%		
6	5	2	0	0	2	0.152%	3.79E-03	0.6
6	6	541	125	649	1315	99.621%		
6	7	1	0	2	3	0.227%		
7	6	0	0	2	2	1.047%	1.05E-02	0
7	7	82	48	59	189	98.953%		
8	8	31	6	10	47	97.917%	2.08E-02	1
8	9	1	0	0	1	2.083%		
9	9	16	20	20	56	100.000%	0	-
10	10	3	2	0	5	62.500%	3.75E-01	1
10	11	1	2	0	3	37.500%		

SUPPLEMENTAL FIGURES

Figure S1. The numbers of STR-containing loci that uniquely mapped to the reference genome using different mappers. One cDNA library from an orangutan testis sample sequenced in one batch was analyzed. The shared uniquely mapped loci are shown as the intersection of all three methods of the Venn diagram. The parameters used were as follow:

bwa aln -n 0 -o 0

tophat --read-mismatches 0 --read-gap-length 0 --read-edit-dist 0 --mate-inner-dist 12 --library-type fr-unstranded

STAR --runThreadN 1 --outFilterType BySJout --outFilterMultimapNmax 1 --alignSJoverhangMin 8 --alignSJDBoverhangMin 1 --outFilterMismatchNmax 0 --alignIntronMin 20 --alignIntronMax 4000 --alignMatesGapMax 1000000

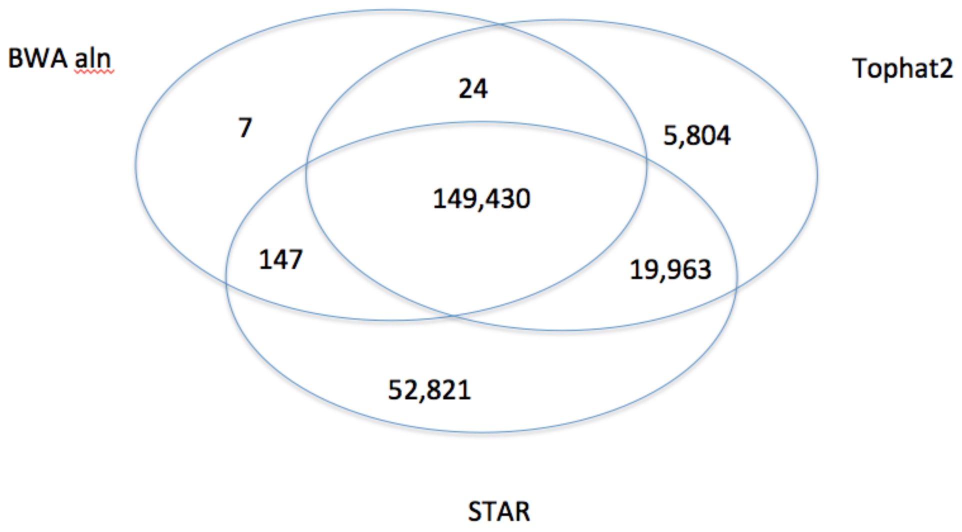

Figure S2. The frequency distribution of the $(A)_n$-containing loci as a function of RNA-seq sequencing depth.

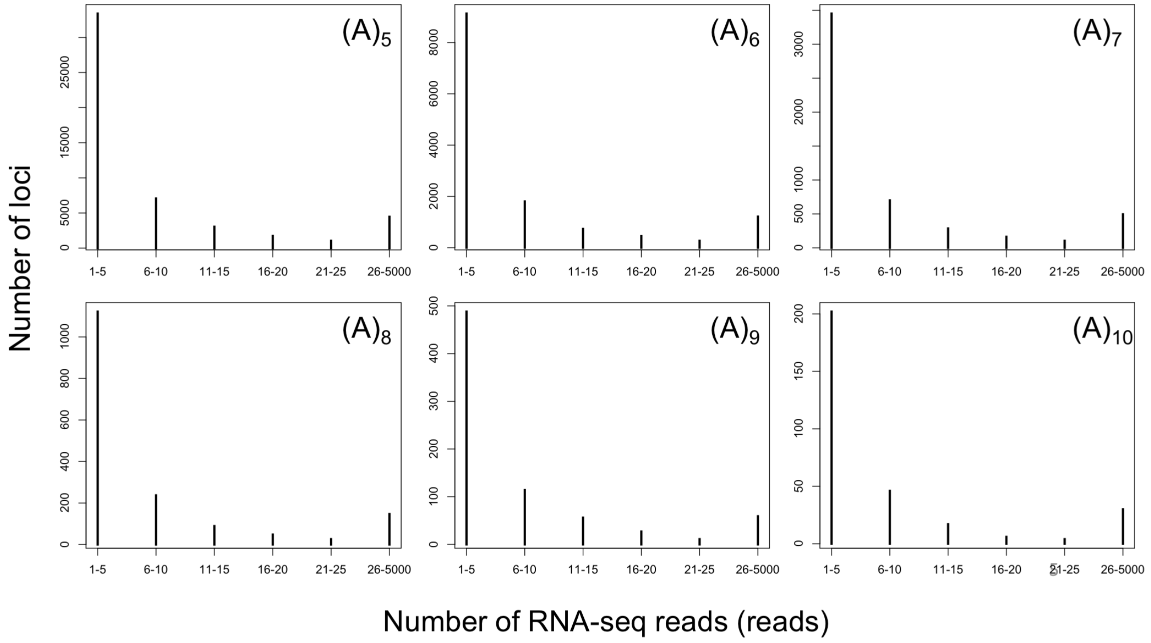

Figure S3. The performance of MLE on simulated data with the bin size of 2; parameters: RDD = 0.05, p_{RDD} = 0.7, RT error = 0.01, p_{RT} = 0.3; 1,000 loci. The red line indicates true parameter values. The circles and the vertical lines show the medians and 95% confidence intervals of the estimated parameters, respectively.

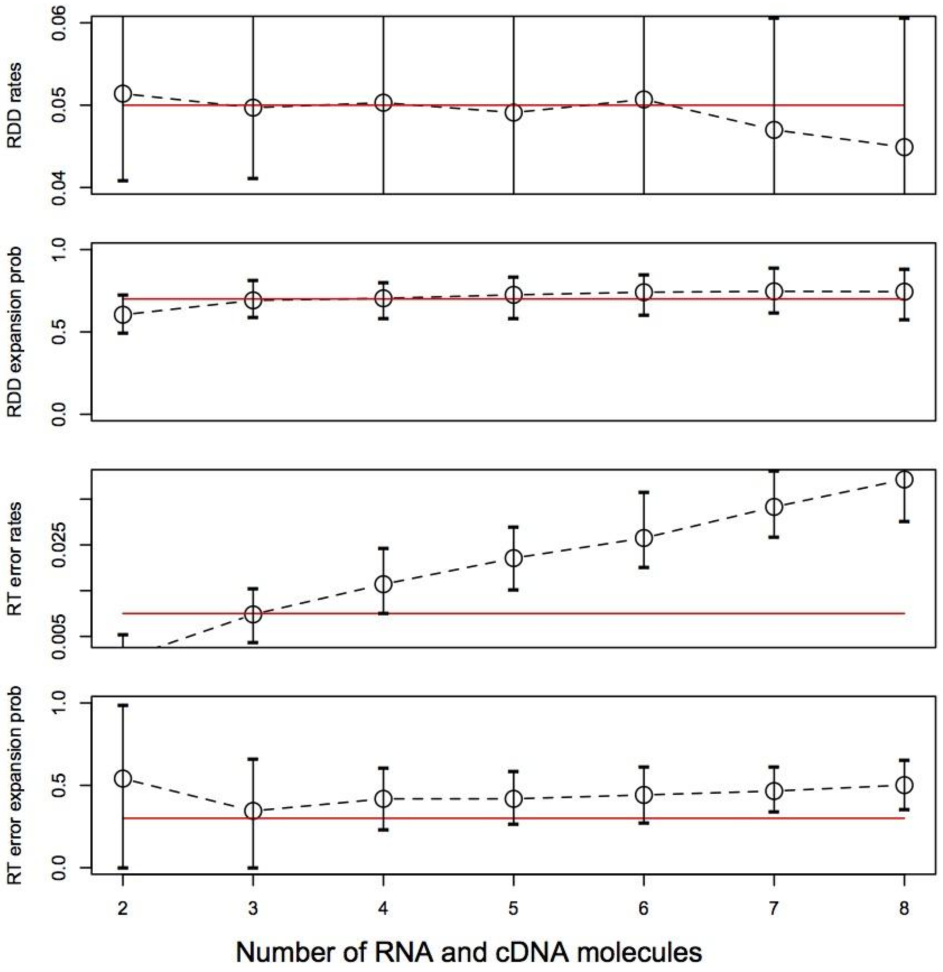

Figure S4.The performance of MLE on simulated data with the bin size of 2; parameters: RDD = 0.01, p_{RDD} = 0.8, RT error = 0.01, p_{RT} = 0.8; 1,000 loci. The red line indicates true parameter values. The circles and the vertical lines show the medians and 95% confidence intervals of the estimated parameters, respectively.

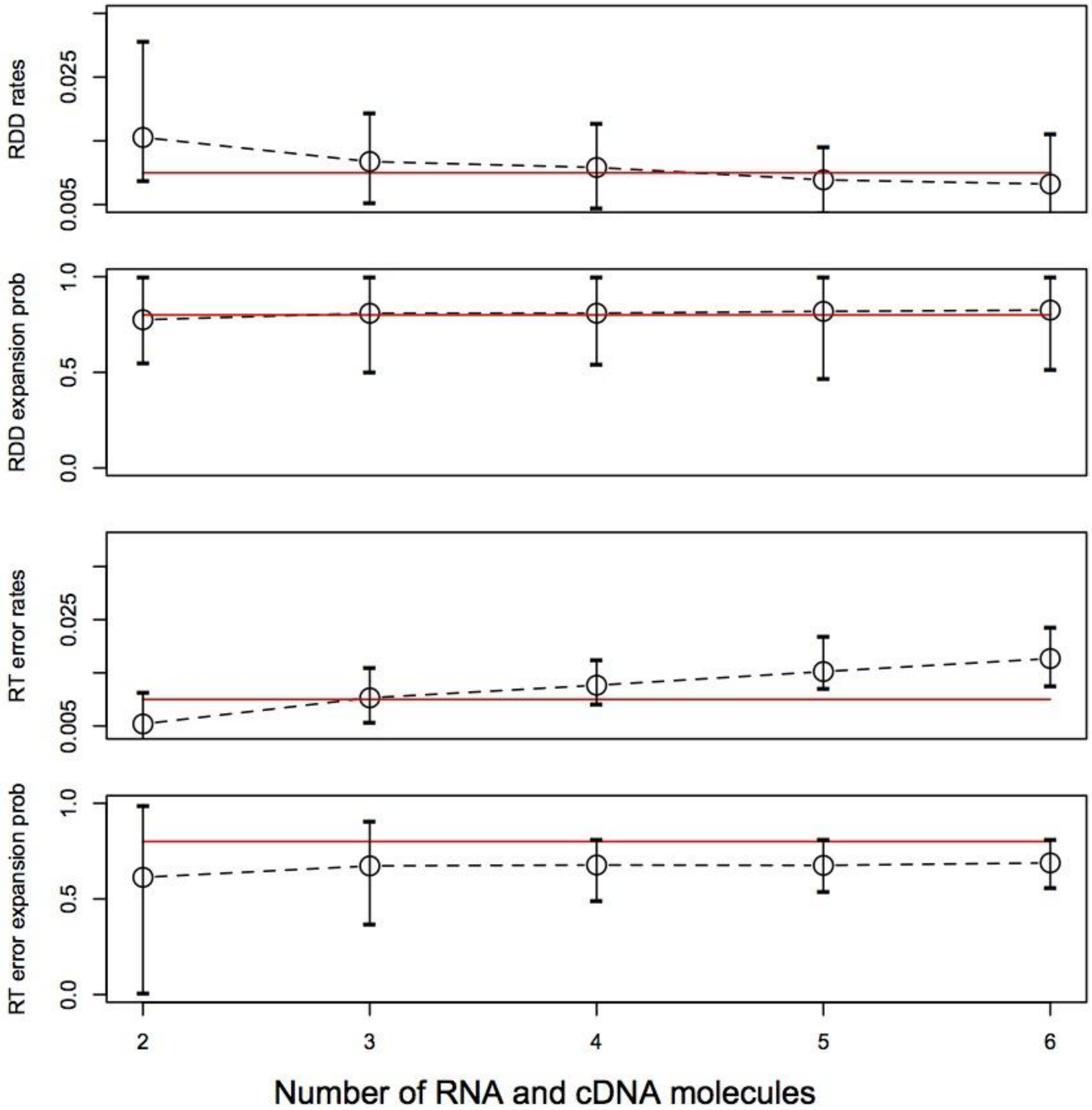

Figure S5. A schema of the barcoded RNA sequencing analysis. At least two reads were used to infer each cDNA molecule. Two to three cDNA molecules (in the punctuated orange rectangle) were used to infer one RNA molecule and its RT errors. RDDs were estimated from loci with at least two RNA families (in the punctuated purple rectangle).

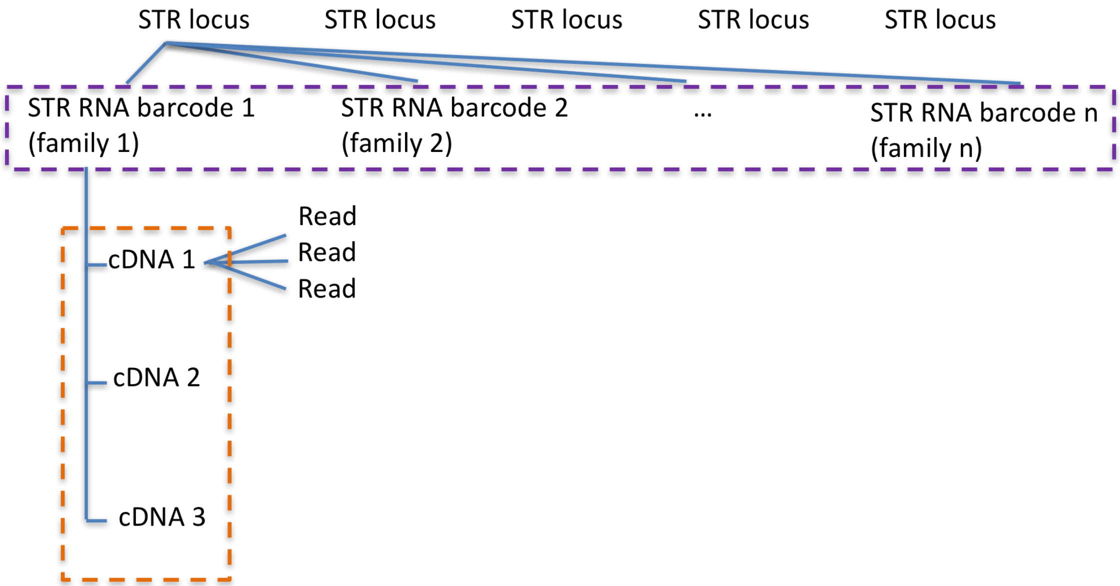

Figure S6. Frequency distribution of the (A)$_n$-containing loci as a function of their repeat number *n* in RNA sequencing data.

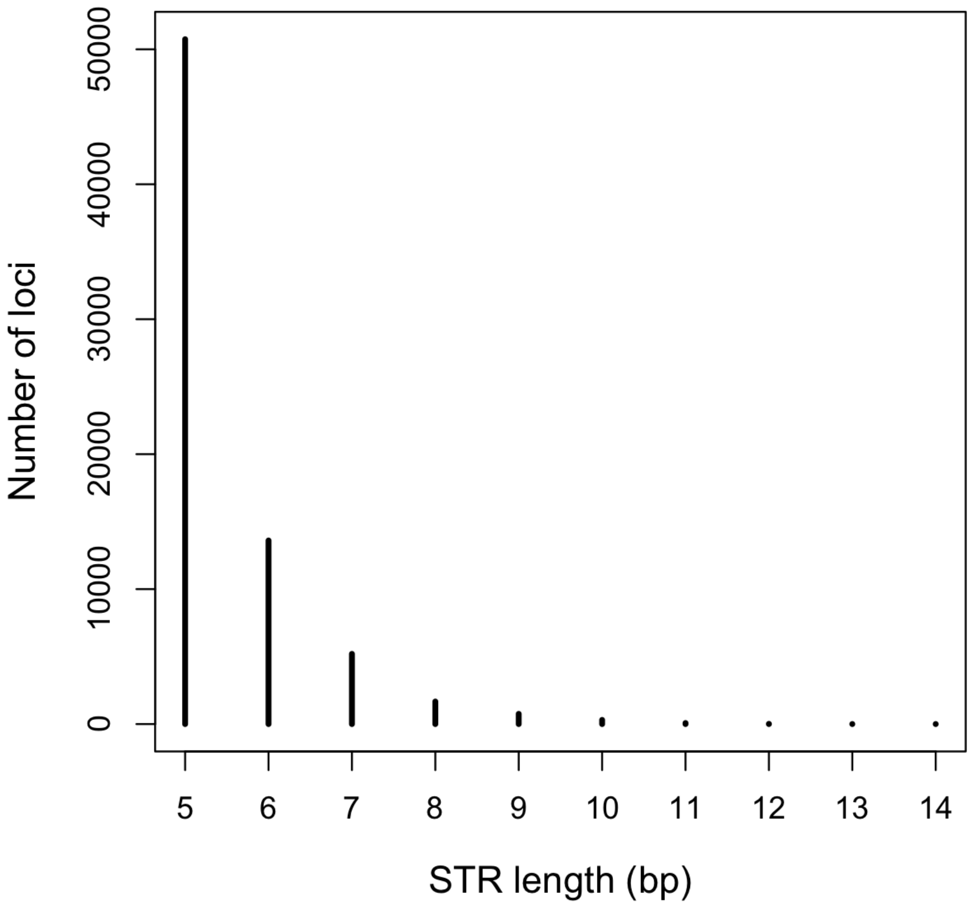

Figure S7. The performance of MLE on simulated data with the bin size of 3; parameters: RDD = 0.05, p_{RDD} = 0.7, RT error = 0.01, p_{RT} = 0.3; 1,000 loci. The red line indicates true parameter values. The circles and the vertical lines show the medians and 95% confidence intervals of the estimated parameters, respectively.

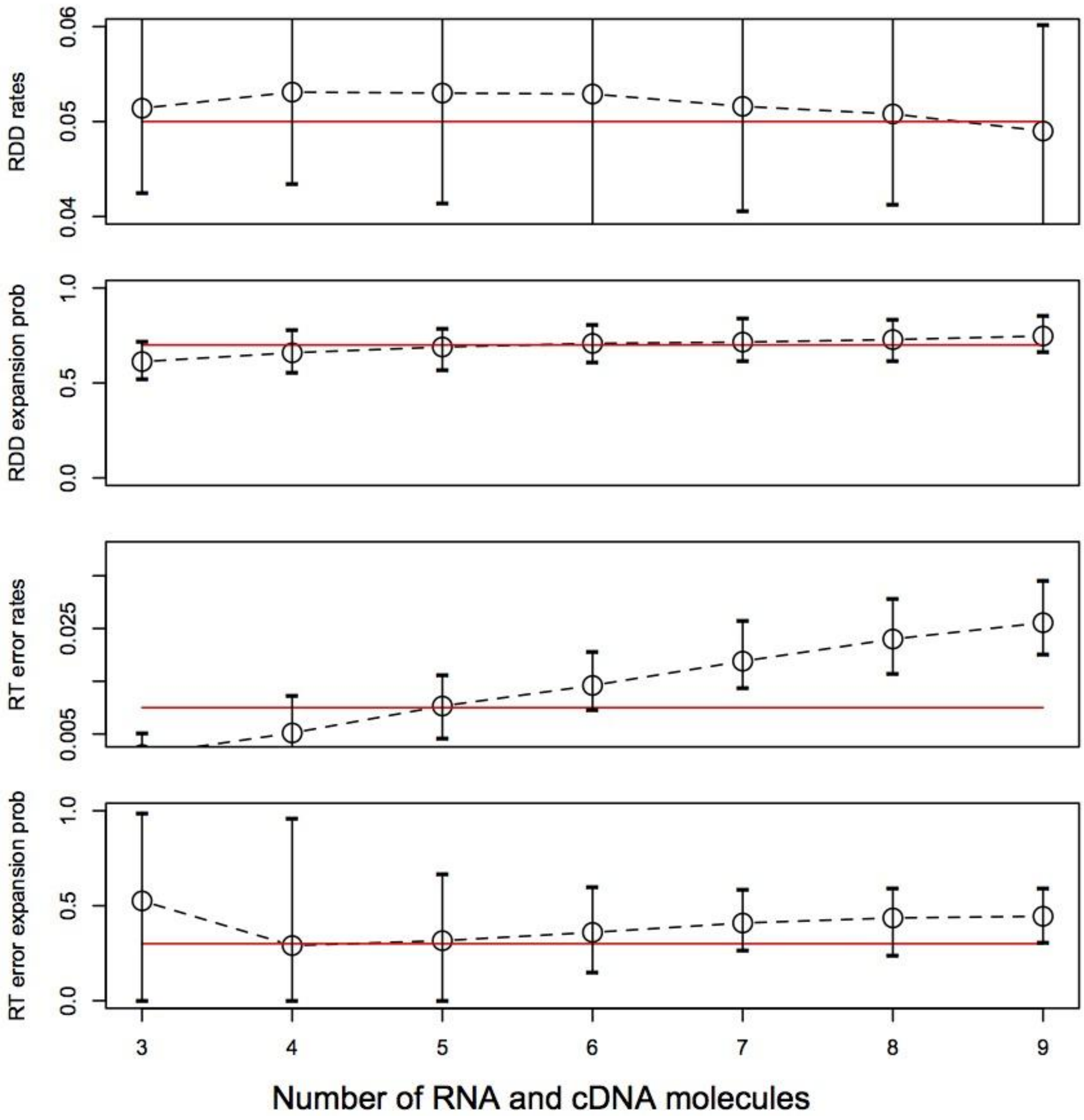

Figure S8. The performance of MLE on simulated data with the bin size of 5; parameters: RDD = 0.05, p_{RDD} = 0.7, RT error = 0.01, p_{RT} = 0.3; 1,000 loci. The red line indicates true parameter values. The circles and the vertical lines show the medians and 95% confidence intervals of the estimated parameters, respectively.

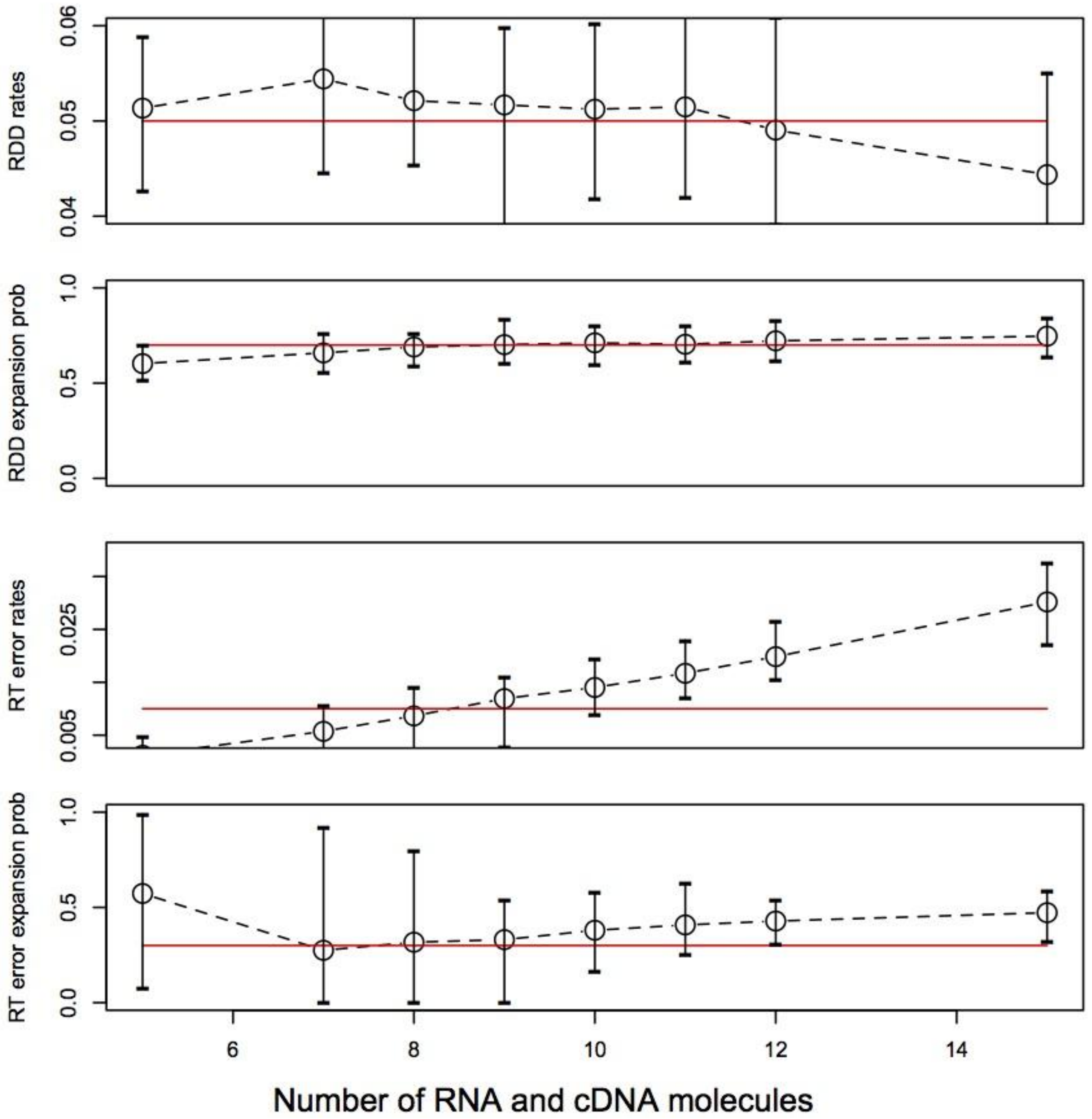

Figure S9. The performance of MLE on simulated data with the bin size of 5; parameters: RDD = 0.01, p_{RDD} = 0.8, RT error = 0.01, p_{RT} = 0.8; 1,000 loci. The red line indicates true parameter values. The circles and the vertical lines show the medians and 95% confidence intervals of the estimated parameters, respectively.

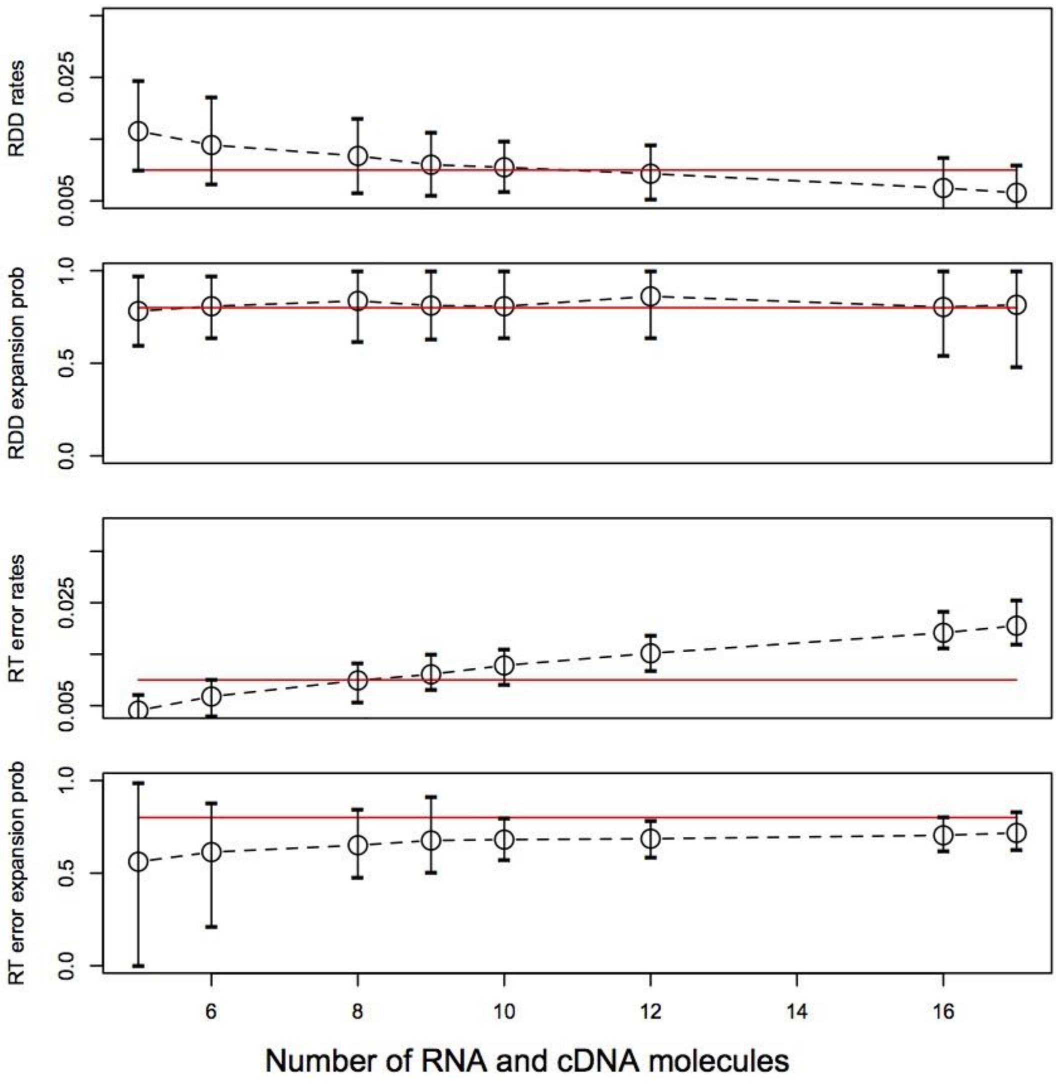

Figure S10. The performance of MLE on simulated data with the bin size of 2; parameters: RDD = 0.01, p_{RDD} = 0.8, RT error = 0.01, p_{RT} = 0.8; and number of RNA and cDNA molecules = 3. The red line indicates true parameter values. The circles and the vertical lines show the medians and 95% confidence intervals of the estimated parameters, respectively.

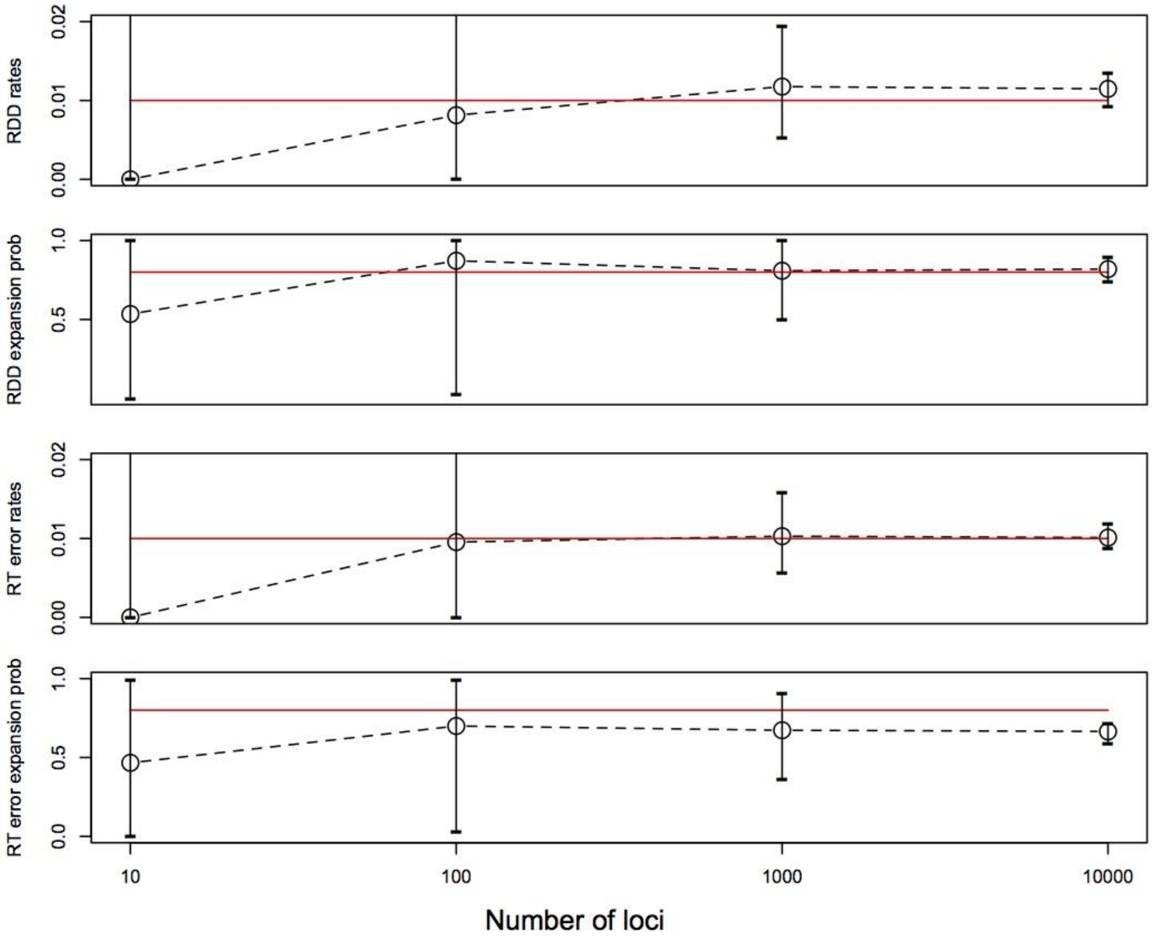

Figure S11. RDD rates, RDD expansion probabilities, RT error rates, and RT expansion probabilities inferred by applying the lumping MLE to all four sequencing runs of orangutan data using bin size of 5 and 6-16 RNA-seq depth of data. Median values across 100 empirical bootstrap replicates (bootstrapped across loci) are plotted with open circles, whereas point estimates are plotted with stars. Solid lines connect the median bootstrap estimates. The 95% confidence intervals were calculated from the 100 bootstraps replicates. RDD was zero at five repeats, therefore RDD expansion probability was not defined for this repeat number.

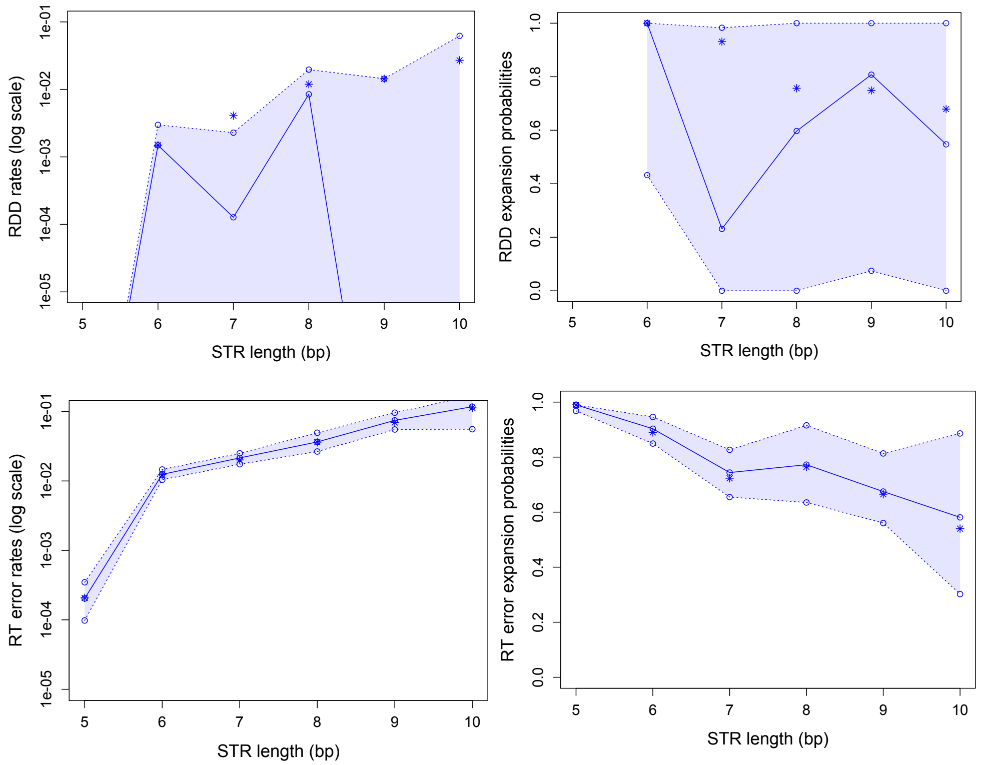

Figure S12. RT error rates inferred by the full MLE for the orangutan data batches A (blue) and B (red) and by the lumping MLE without using barcode information (black) for *C. elegans* data as a function of repeat number for $(A)_n$. The three black lines represent estimates from the wild-type strain, the VC1305 line, and the RB886 line. Median values across 100 empirical bootstrap replicates (bootstrapped across loci) are plotted with open circles, whereas point estimates are plotted with stars. Solid lines connect the median bootstrap estimates. The 95% confidence intervals were calculated from the 100 bootstraps replicates.

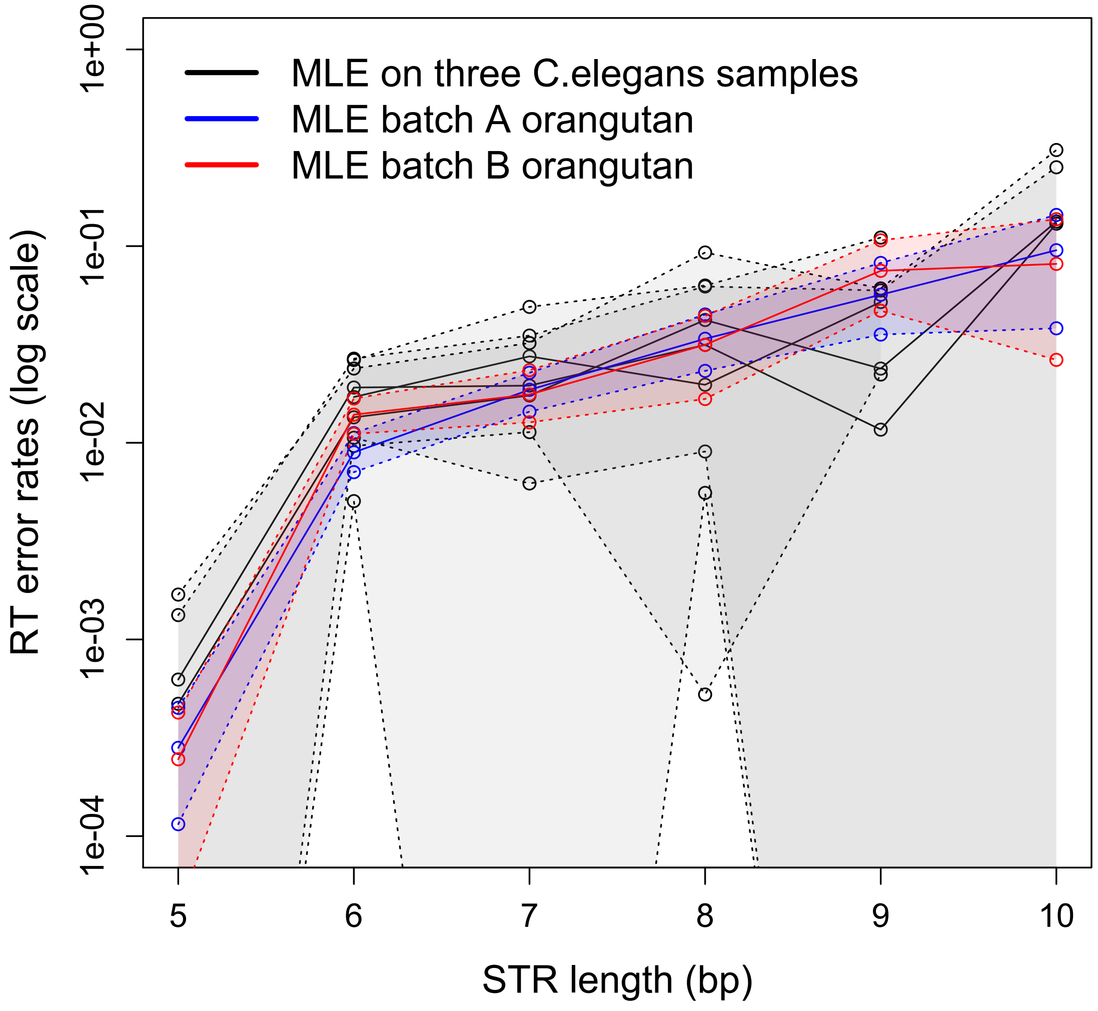

Supplement: Supplementary Data [file supp_msw139_suppl_data.zip › Supplemental_Figs_Tables_June8.pdf]
